# Dual Gut Microbiota‐Dependent Mechanisms Underlying the Anti‐Atherosclerotic Effect of Tilianin

**DOI:** 10.1002/mco2.71141

**Published:** 2026-11-08

**Authors:** Xuli Ruan, Ruyue Liu, Wenjiang Cao, Liping Wang, Mengran Guo, Zhongshan He, Yupei Zhang, Yaoyao Luo, Yuntao Gao, Chuansheng Huang, Yunli Zhao, Yong Yuan, Xiangrong Song, Xinchun Wang

**Affiliations:** ^1^ Department of Pharmacy First Affiliated Hospital of Shihezi University Shihezi China; ^2^ Key Laboratory of Xinjiang Phytomedicine Resource and Utilization School of Pharmacy Ministry of Education Shihezi University Shihezi China; ^3^ Department of Clinical Pharmacy Department of Critical Care Medicine State Key Laboratory of Biotherapy and Cancer Center West China Hospital Sichuan University Chengdu China; ^4^ Department of Head and Neck Surgery Sichuan Clinical Research Center for Cancer Sichuan Cancer Center Sichuan Cancer Hospital & Institute University of Electronic Science and Technology of China Chengdu China

**Keywords:** atherosclerosis, gut microbiota, metabolism, tilianin, TMA/TMAO

## Abstract

Tilianin (Til), a flavonoid from *Dracocephalum moldavica* L. and *Agastache* species, exerts potent anti‐atherosclerotic effects via gut microbiota‐mediated mechanisms. In atherosclerotic mice, Til dose‐dependently reduced plaque burden and enhanced plaque stability by reducing necrotic cores and increasing collagen. Til also improved lipid profiles, attenuated inflammation, and lowered trimethylamine N‐oxide (TMAO) levels, accompanied by microbiota remodeling—specifically, enhancing α‐diversity and enriching *Akkermansia muciniphila*. Fecal microbiota transplantation from Til‐treated donors successfully transferred these protective phenotypes. Conversely, antibiotic‐induced microbiota depletion selectively abrogated the plaque‐stabilizing arm of Til's action, leaving its effect on overall plaque burden intact, despite maximized TMAO reduction and increased systemic exposure. This “antibiotic paradox” stemmed from short‐chain fatty acid (SCFA) depletion and intestinal barrier breakdown (decreased ZO‐1/occludin), triggering metabolic endotoxemia (elevated LPS). Mechanistically, Til directly inhibits microbial trimethylamine monooxygenase (TMM) with nanomolar potency (IC_50_ = 18.78 nM), blocking local TMA‐to‐TMAO conversion. Overall, Til's lipid‐lowering, anti‐inflammatory, and antioxidant activities in vivo are gated by a pivotal microbiota–metabolite–barrier axis, acting through dual enzymatic inhibition and homeostasis maintenance, offering novel insights for flavonoid‐based multi‐target atherosclerosis therapies.

## Graphical abstract

(A) Tilianin reshapes the gut microbiota by increasing Bacteroidota, *Akkermansia muciniphila*, short‐chain fatty acids, and gut‐barrier integrity, while reducing Firmicutes, TMA, and TMAO. Gut microbial enzymes transform tilianin into acacetin and acacetin‐7‐O‐glucuronide. (B) These changes lower plasma LPS, improve lipid profiles and inflammatory cytokines, promote M1‐to‐M2 macrophage polarization, reduce ROS, and alleviate atherosclerotic plaque burden.

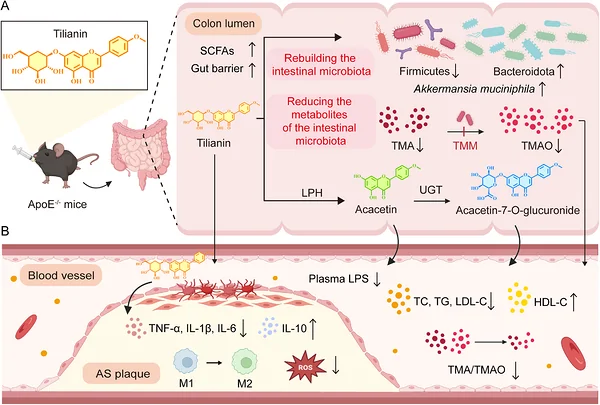

## 1 Introduction

Cardiovascular disease (CVD) causes approximately 19.8 million deaths annually worldwide, and ischemic heart disease and stroke—both driven principally by the pathological process of atherosclerosis (AS)—together account for approximately 85% of these deaths [1, 2, 3, 4]. While statins remain the first‐line therapy, their use is often limited by intolerance and adverse effects [5, 6], highlighting the urgent need for safer, multitarget agents derived from medicinal food plants [7, 8]. *Agastache* species (e.g., *A. rugosa*, *A. mexicana*) and *Dracocephalum moldavica* L. are representative medicinal‐edible plants traditionally integrated into both diets and therapeutic systems under the concept of “medicine‐food homology” [9, 10, 11, 12]. The tender stems, leaves, and flowers of these plants are frequently consumed as functional teas or foods due to their appealing taste and documented health benefits. Phytochemical studies have identified rich profiles of phenolics, flavonoids, and terpenoids in these edible parts [13, 14, 15]. Among these, tilianin (Til), a flavonoid glycoside, has emerged as a principal bioactive compound consistently found across all three species [16, 17, 18].

Both in vitro and in vivo studies have demonstrated that Til exerts significant anti‐atherosclerotic effects through multiple mechanisms, including suppression of vascular inflammation, reduction of oxidative stress, regulation of lipid metabolism, and modulation of macrophage polarization—all pivotal events in atherogenesis [19, 20]. These pleiotropic actions position Til as a promising candidate for the prevention and treatment of AS. However, the low bioavailability of Til significantly limits its clinical potential, drawing increasing attention from researchers aiming to overcome this barrier. Pharmacokinetic analyses indicate that Til undergoes extensive metabolic transformation in vivo. Our previous research identified two principal plasma metabolites: acacetin (Aca) and its conjugate acacetin‐7‐O‐glucuronide (Aca‐7‐G). Notably, gut microbiota‐derived β‐glucuronidase (BGUS) catalyzes the deconjugation of Aca‐7‐G, regenerating Aca and facilitating its reabsorption through enterohepatic circulation—thus enhancing systemic exposure to Til‐derived metabolites [21]. In addition, Til supplementation increased the abundance of beneficial microbes in ApoE^−/−^ mice [22]. These findings point to a dual role of gut microbiota in both regulating Til metabolism and mediating its anti‐atherosclerotic efficacy. This suggests a pivotal role of the gut microbiota in determining Til's bioavailability and pharmacodynamic activity.

Comprising over 100 trillion microorganisms—predominantly Bacteroidetes, Firmicutes, and Actinobacteria—the gut microbiome influences AS through several interconnected mechanisms [23, 24, 25, 26, 27, 28]. The gut microbiota, known as the host's “second genome,” plays a key role in xenobiotic metabolism [29]. It influences drug bioavailability, efficacy, and toxicity through enzymes [30]. Accumulating evidence indicates that the gut microbiota is a key modulator of AS pathogenesis. Beneficial metabolites like short‐chain fatty acids (SCFAs) exert anti‐inflammatory effects and improve vascular function [31, 32]. Conversely, microbial processing of dietary choline/carnitine generates trimethylamine (TMA) and trimethylamine N‐oxide (TMAO), promoting foam cell formation and plaque instability [33, 34, 35]. In addition, microbial components (e.g., lipopolysaccharide [LPS]) activate pro‐inflammatory pathways. These dual roles establish the gut microbiota as a promising therapeutic target for AS [36].

In the present study, we systematically investigated the gut microbiota‐mediated mechanisms underlying the anti‐atherosclerotic efficacy of Til. We first established a clear dose–response relationship for Til in ApoE^−/−^ mice and employed fecal microbiota transplantation (FMT) and antibiotic‐induced microbiota depletion to validate the pivotal role of the microbiome. To resolve the pharmacokinetic–pharmacodynamic (PK–PD) paradox—where systemic drug exposure increased but efficacy was lost following microbiota depletion—we performed integrated profiling of the intestinal barrier integrity and microbiota‐derived metabolites, such as SCFAs and LPS. Finally, to pinpoint the direct molecular targets, we utilized molecular docking combined with in vitro whole‐cell functional assays to evaluate the capacity of Til and its derivatives to suppress microbial TMA‐to‐TMAO conversion via the trimethylamine monooxygenase (TMM) enzyme. This study provides a comprehensive framework for understanding how Til achieves multi‐pathway protection by balancing the microbiota–metabolite–barrier axis.

## 2 Results

### 2.1 The Gut Microbiota Remodeled by Til Conferred Anti‐Atherosclerotic Effects

To determine the optimal therapeutic dosage and validate the dose–response relationship of Til, we conducted additional experiments with varying doses (2.1, 3.5, and 7 mg/kg/d) and included simvastatin (3.5 mg/kg/d) as a positive control. Quantitative analysis of the whole aorta and aortic root sections revealed a clear dose‐dependent reduction in plaque area and improvement in plaque stability (Figure S1). Specifically, the high‐dose group (Til‐H, 7 mg/kg/d) exhibited the most robust anti‐atherosclerotic efficacy, which was comparable to the therapeutic effect of simvastatin. Based on these dose–response findings, 7 mg/kg/d was selected as the optimal concentration for subsequent mechanistic investigations.

The therapeutic effects of Til and gut microbiota modulation on AS were assessed by evaluating aortic plaque burden and vascular histopathology. The schematic of the dosing regimen in mice is shown in Figure 1A. As shown in Figure 1B, C, representative images of Oil Red O (ORO) staining and corresponding quantitative analysis revealed that the lipid‐stained area in the model group reached 61.4 ± 10.3%, significantly higher than in the control group, confirming successful establishment of the AS model. Treatment with Til or FMT significantly reduced the stained area compared with the model group, indicating attenuation of plaque formation. Although the A and AT groups also showed reductions in plaque area, the differences were not statistically significant. Notably, plaque area in the AT group did not differ from that in the Til group (*p* = 0.999), indicating that antibiotic co‐treatment did not abolish the effect of Til on overall plaque burden. These findings suggest that both Til and its microbiota remodeling effects contribute to reducing aortic plaque burden.

**FIGURE 1:**
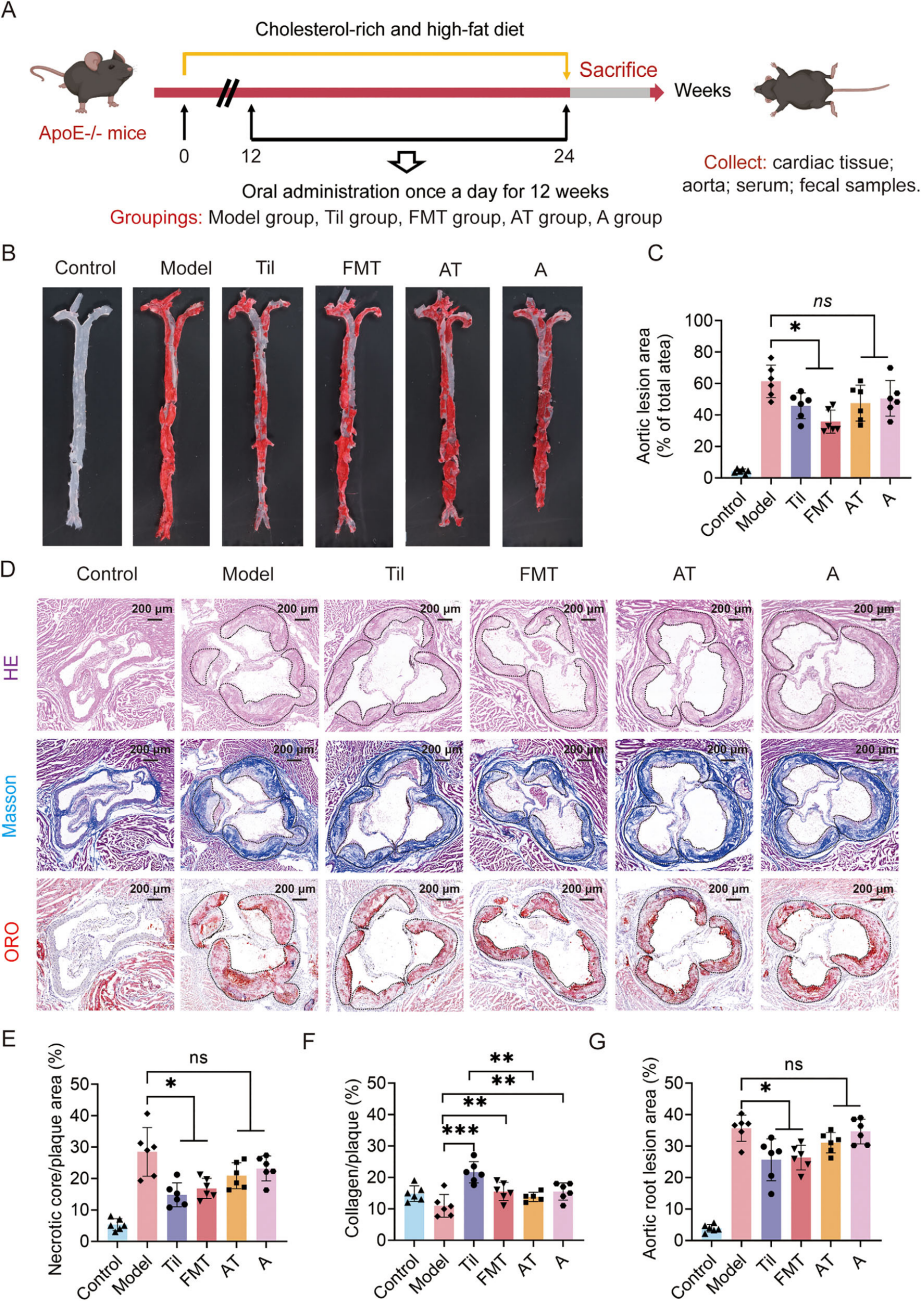
Anti‐atherosclerotic effects of gut microbiota modulated by Til. (A) Schematic of the experimental timeline: drug administration and sample collection in mice. Model group: maintain the saline treatment; Til group: 7 mg/kg tilianin treatment; FMT group: fecal suspension from the Group Til, administered after the antibiotic pretreatment described in Section 5; AT group: quadruple antibiotic + 7 mg/kg tilianin treatment; A group: quadruple antibiotic treatment. (B and C) Representative images and quantification of Oil Red O (ORO) staining of whole aortas. (D–G) Representative images and quantification of H&E, Masson, and ORO staining in aortic root sections. Scale bar = 200 µm. Data are expressed as mean ± SD (*n* = 6). ^*^ *p* < 0.05 versus Model; ^**^ *p* < 0.01 versus Model; *** p < 0.001 versus Model; ns, not significant versus Model.

Histological examination of the aortic root further supported these results. As shown in Figure 1D–G, hematoxylin and eosin (H&E) staining revealed extensive necrotic cores and cholesterol crystals in the model group plaques. In contrast, the necrotic core areas were significantly reduced in the Til, FMT, and AT groups, while the A group showed a decreasing trend without statistical significance. Masson's trichrome staining demonstrated that collagen content was markedly increased in plaques of the Til group compared with the model group, suggesting enhanced plaque stability; more modest increases were observed in the FMT and A groups, whereas the AT group did not differ from the model group. Critically, collagen content in the AT group was significantly lower than in the Til group (*p* = 0.001), whereas necrotic core area and lesion area did not differ between these two groups (*p* = 0.208 and *p* = 0.999), indicating that antibiotic co‐treatment selectively removed the plaque‐stabilizing component of Til's action. In aortic root sections stained with ORO, consistent with whole‐aorta findings, the Til and FMT groups exhibited significantly reduced plaque areas compared with the model group, whereas the A and AT groups showed no significant changes.

Serum lipid profiles were analyzed to assess lipid metabolism. As shown in Figure 2A–D, the model group exhibited significantly increased serum levels of total cholesterol (TC), triglycerides (TG), and low‐density lipoprotein cholesterol (LDL‐C), and decreased high‐density lipoprotein cholesterol (HDL‐C) compared to the control group. Til treatment significantly lowered TG and raised HDL‐C (*p* = 0.019 and *p* < 0.001 vs. Model); TC and LDL‐C fell by a comparable proportion but did not reach significance once the unequal variances of these two measures were taken into account (*p* = 0.143 and *p* = 0.163, Tamhane's T2). Notably, the AT group also showed significant improvement in TG and HDL‐C (*p* < 0.001 and *p* = 0.006 vs. Model), although its HDL‐C remained significantly below that of the Til group (*p* = 0.009). The FMT group showed the same trends without reaching significance, and no significant changes in lipid levels were observed in the A group.

**FIGURE 2:**
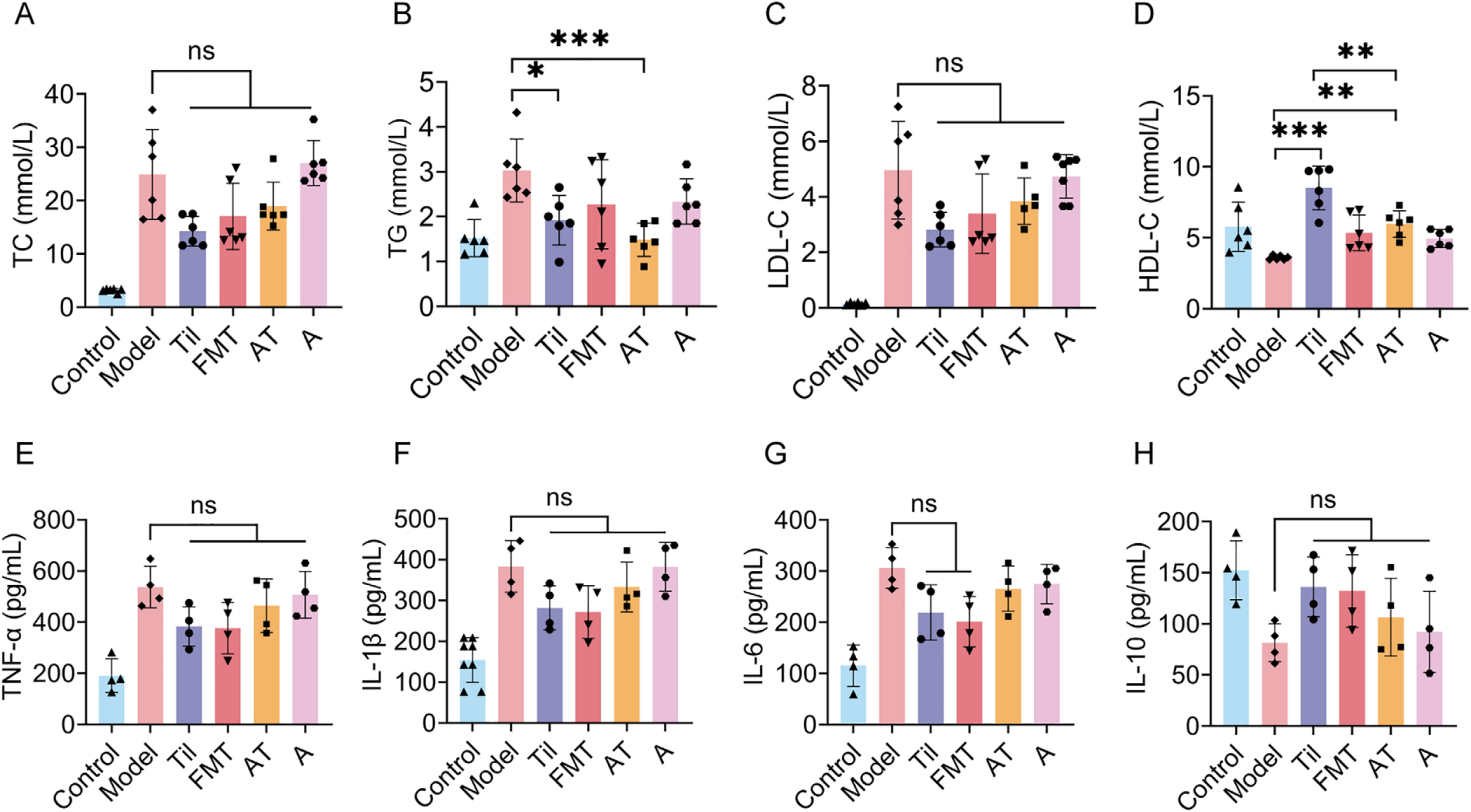
The gut microbiota remodeled by Til ameliorated serum lipid profiles and attenuated systemic inflammation. (A–D) Serum levels of TC, TG, LDL‐C, and HDL‐C (*n* = 6). (E–H) Serum levels of TNF‐α, IL‐1β, IL‐6, and IL‐10. Data are expressed as mean ± SD (*n* = 6 for A–D; *n* = 4 for E–H, see Section 5). ^*^ *p* < 0.05; ^**^ *p* < 0.01; ^***^ *p* < 0.001 vs. Model; ns, not significant versus Model.

Systemic inflammation was evaluated by measuring serum inflammatory cytokines. As illustrated in Figure 2E–H, the model group showed significant elevations in interleukin (IL)‐6, tumor necrosis factor‐alpha (TNF‐α), and IL‐1 beta (IL‐1β), along with reduced IL‐10 levels compared to the control group. Til and FMT significantly reduced IL‐6 (*p* = 0.050 and *p* = 0.015 vs. Model). Changes in the same direction were observed for the remaining cytokines in both groups—TNF‐α fell by 29% and 30%, IL‐1β by 27% and 29%, and IL‐10 rose by 67% and 62% relative to the model group—but with four animals per group these differences did not reach statistical significance (*p* = 0.065–0.143); the in vitro macrophage experiments described below address the anti‐inflammatory activity of Til and its metabolites directly. No significant changes in cytokine levels were observed in the A or AT groups.

### 2.2 PGF Increases the Total Plasma Exposure of Til and Its Metabolites

Given that gut microbiota‐derived metabolic enzymes participate in the in vivo disposition of Til, a pseudo‐germ‐free (PGF) mouse model was established to assess the impact of microbiota depletion on its pharmacokinetics. To verify the effectiveness of microbiota depletion, fecal samples were collected and cultured under anaerobic conditions to quantify bacterial colonies. As shown in Figure S2, colony‐forming unit (CFU) counts in the PGF group were significantly lower than those in the control group, confirming successful establishment of the PGF model.

After a single oral administration of Til at either low or high doses, the parent compound (Til), its aglycone metabolite (Aca), and its glucuronide conjugate (Aca‐7‐G) were detected in plasma samples. The corresponding plasma concentration–time profiles are shown in Figure 3A and Figure S3A, and key pharmacokinetic parameters are summarized in Tables S1 and S2. The AUC_0‐t_ statistical plots are shown in Figure 3B and Figure S3B. At the low dose, the AUC_0‐t_ of Til in the PGF group was 4.0 times that in the control group (3447.2 ± 1352.0 vs. 870.3 ± 654.3 µg·min/L, *p* < 0.05). At the high dose, the corresponding value was 1.9 times that in the control group (8588.5 ± 6217.8 vs. 4467.5 ± 616.2 µg·min/L). In contrast, the AUC_0‐t_ of Aca in the PGF group was 27.5% and 33.3% lower than that in the control group at the low and high doses, respectively. The AUC_0‐t_ of Aca‐7‐G in the PGF group was 1.5 and 3.3 times that in the control group at the low and high doses, respectively.

**FIGURE 3:**
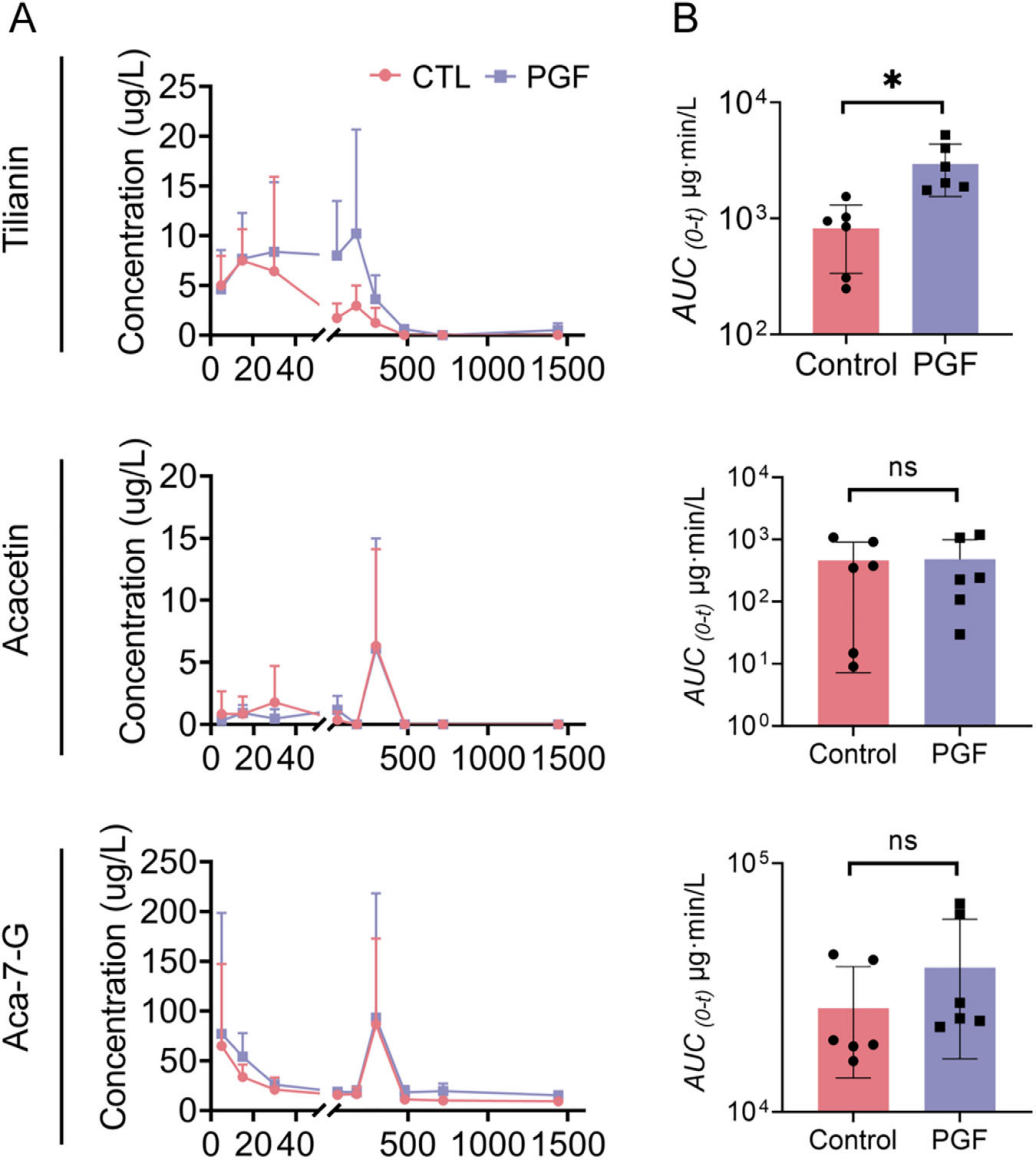
Effect of gut microbiota on the pharmacokinetics of Til and its metabolites. (A) Plasma concentration–time profiles of Til, Aca, and Aca‐7‐G after oral administration of Til (7 mg/kg) in conventional and PGF mice. (B) AUC_0‐t_ of Til, Aca, and Aca‐7‐G after oral administration of Til (7 mg/kg) in conventional and PGF mice. Data are expressed as mean ± SD (*n* = 6). ^*^ *p* < 0.05; ns, not significant.

In summary, these results indicate that antibiotic treatment significantly increased the overall plasma exposure of Til and its metabolites in C57BL/6J mice.

### 2.3 Til and Its Metabolites Both Have Anti‐Inflammatory and Antioxidant Activities In Vitro

To assess the in vitro anti‐inflammatory and antioxidant activities of Til and its metabolites, a macrophage inflammation model was established. Flow cytometry was employed to evaluate macrophage polarization by quantifying the proportions of M1 and M2 macrophages. As shown in Figure 4A–D, the model group exhibited a marked increase in M1 macrophages and the M1/M2 ratio, confirming successful induction of inflammation. Til treatment significantly reduced the proportion of M1 macrophages compared to the Model, Aca, and Aca‐7‐G groups, while all three treatment groups (Til, Aca, and Aca‐7‐G) significantly increased the proportion of M2 macrophages and decreased the M1/M2 ratio compared to the model group.

**FIGURE 4:**
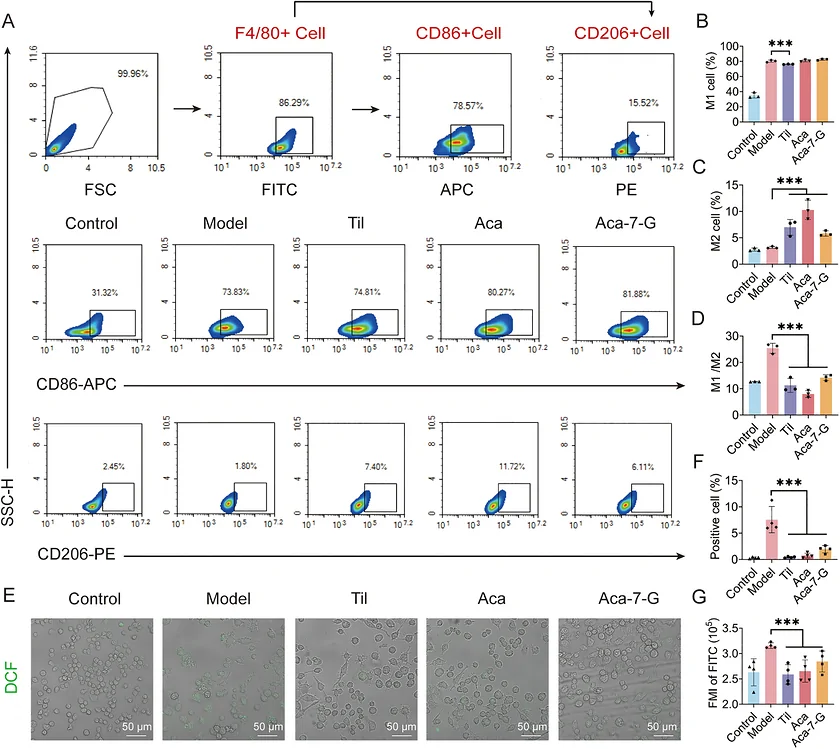
In vitro anti‐inflammatory and antioxidant effects of Til and its metabolites. (A) Gating strategy and representative flow cytometry plots. (B–D) Quantification of the proportions of M1 and M2 macrophages and the M1/M2 ratio in each group. (E) Representative fluorescence images of intracellular ROS (DCFH‐DA staining; green) in RAW264.7 cells. Scale bar = 50 µm. (F and G) Flow cytometry analysis and quantification of ROS‐positive cells after different treatments. Data are shown as mean ± SD (*n* = 3). ^***^ *p* < 0.001.

To evaluate antioxidant effects, intracellular ROS levels were measured using flow cytometry and laser scanning confocal microscopy. As shown in Figure 4E–G, all treatment groups significantly reduced both the proportion of ROS‐positive cells and ROS fluorescence intensity compared to the model group, indicating effective ROS scavenging.

### 2.4 Til Restored the Diversity and Homeostasis of the Gut Microbiota

Microbial diversity analysis revealed that AS progression in ApoE^−/−^ mice was associated with significant gut dysbiosis. The model group showed markedly reduced α‐diversity, as indicated by lower Shannon and Simpson indices compared to the control group (Figure 5A, B), suggesting a decline in microbial richness and evenness. Treatment with Til or FMT significantly restored these indices, while antibiotic treatment led to near‐complete depletion of microbial diversity, validating the dysbiosis model. β‐diversity analysis further confirmed shifts in microbial composition. Principal coordinate analysis (PCoA) and non‐metric multidimensional scaling (NMDS) (Figure 5C, D) revealed distinct clustering of the control group apart from ApoE^−/−^ mice. The Model, Til, and FMT groups formed a closer cluster, indicating partial restoration of community structure by Til and FMT. In contrast, antibiotic‐treated groups exhibited substantial separation, reflecting a profound shift in microbial community profiles.

**FIGURE 5:**
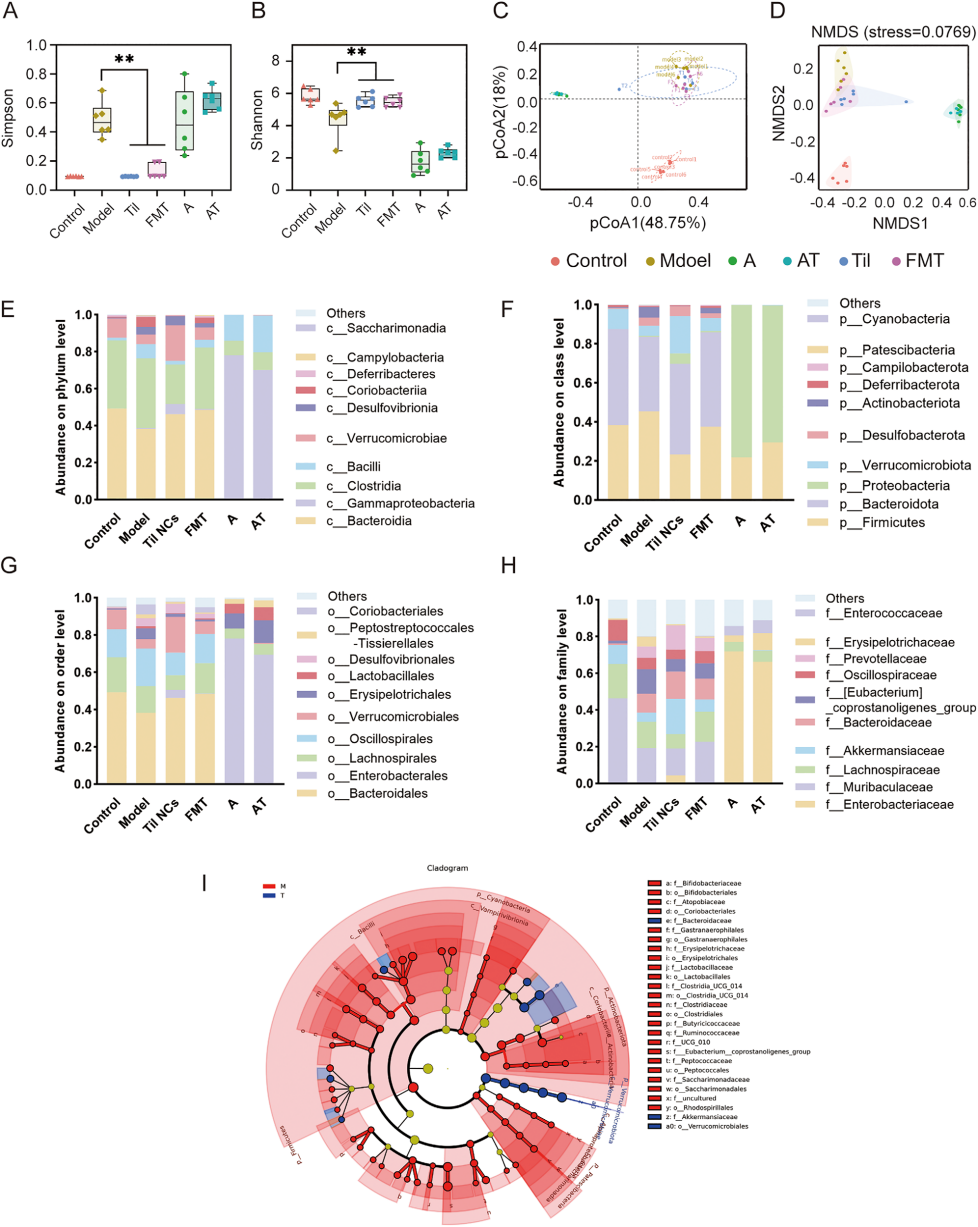
Modulatory effect of Til on gut microbiota composition. (A and B) α‐diversity indices in each group. (C and D) β‐diversity analysis (PCoA and NMDS) in each group. (E–H) Relative abundance of Firmicutes and Bacteroidota and the Firmicutes/Bacteroidota ratio in each group. (I) Differential species comparison between the Til‐ and FMT‐treated groups and the model group. Data are expressed as mean ± SD (*n* = 6). ^**^ *p* < 0.01.

Notable alterations in microbial composition were observed following Til and FMT treatment. In the Til group, the relative abundance of Firmicutes significantly decreased, whereas Bacteroidota increased, resulting in a significantly lower Firmicutes/Bacteroidota (F/B) ratio compared to the model group. A similar trend was observed in the FMT group (Figure 5E–H). In addition, Verrucomicrobiota, particularly the beneficial bacterium *Akkermansia muciniphila*, was significantly enriched in both the Til and FMT groups (Figure 5I). In contrast, the gut microbiota of antibiotic‐treated mice was severely imbalanced, characterized by the dominance of *Escherichia‐Shigella*, indicative of antibiotic‐induced overgrowth of opportunistic pathogens.

Collectively, these results demonstrate that Til administration not only restores gut microbial diversity but also reshapes microbiota composition toward a healthier profile, which may contribute to its protective effects against AS.

### 2.5 Til Attenuates Metabolic Endotoxemia and AS by Restoring SCFAs and Intestinal Barrier Integrity

As shown in Figure 6A–D, while the model group exhibited significantly elevated plasma and fecal levels of TMAO and TMA, Til treatment and FMT successfully suppressed these pro‐atherogenic markers. Notably, although antibiotic treatment (A and AT groups) resulted in the most dramatic reduction of TMAO/TMA, it simultaneously led to a near‐complete depletion of beneficial SCFAs. Specifically, the levels of butyrate and total SCFAs in both plasma and feces were profoundly reduced in the antibiotic‐treated groups (Figure 6E–H).

**FIGURE 6:**
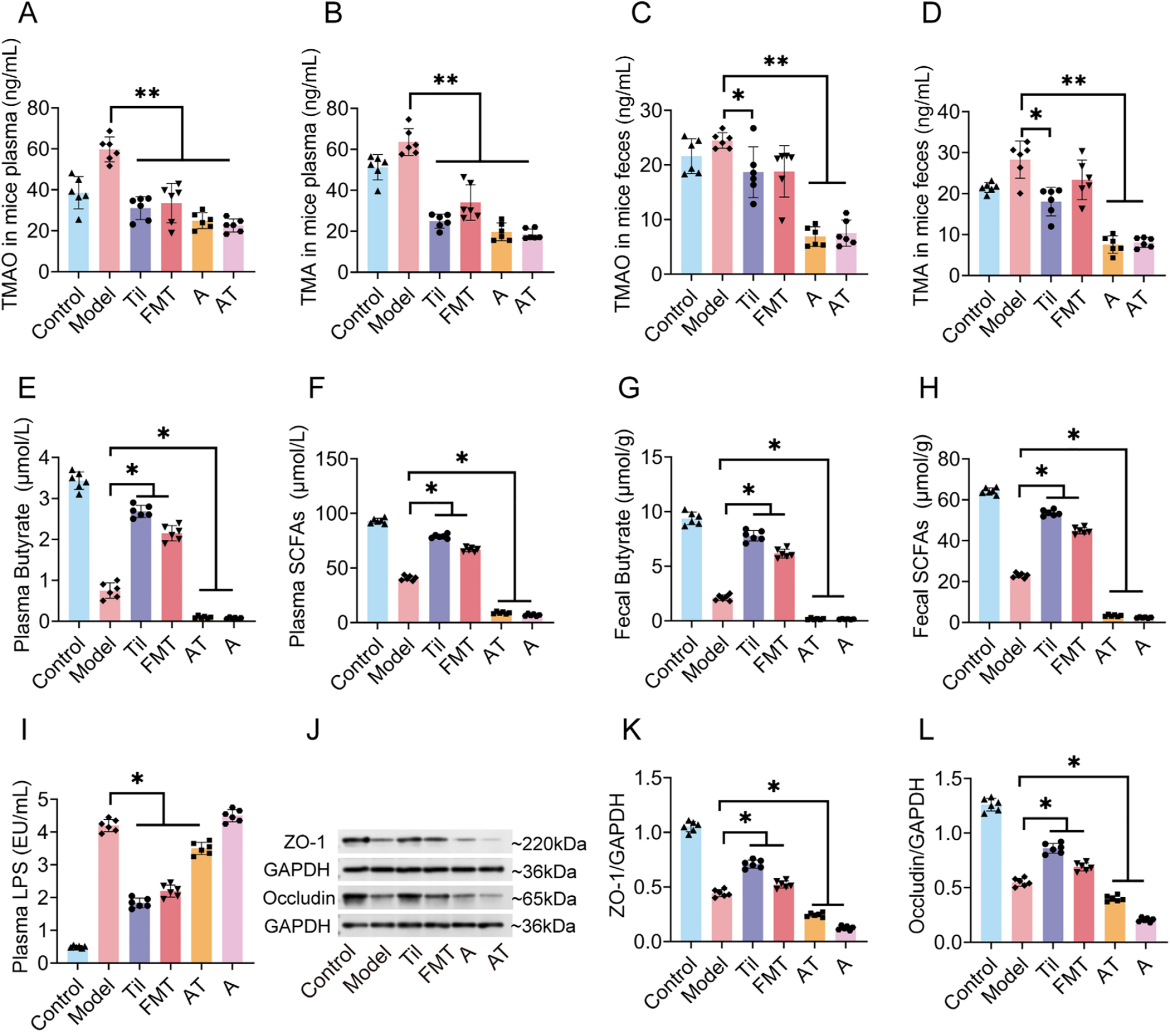
Til modulates gut microbiota‐derived metabolites and restores intestinal barrier integrity. (A–D) Concentrations of TMAO and TMA in (A and B) mice plasma and (C and D) feces across different groups. (E–H) Levels of butyrate and total short‐chain fatty acids (SCFAs) in (E and F) mice plasma and (G and H) feces. (I) Plasma levels of lipopolysaccharide (LPS), indicating metabolic endotoxemia. (j) Representative Western blot images of tight junction proteins (ZO‐1 and occludin) in ileal tissues, with GAPDH as the loading control. (K and L) Quantitative densitometric analysis of (K) ZO‐1 and (L) occludin protein expression normalized to GAPDH. Data are presented as mean ± SD (*n* = 6). ^**^ *p* < 0.01. versus Model.

In contrast, Til and FMT treatment significantly restored SCFA production, particularly butyrate, suggesting that Til preserves the metabolic benefits of the microbiota that are lost during broad‐spectrum antibiotic intervention. Since SCFAs are critical for maintaining gut homeostasis, we next assessed the intestinal barrier integrity. The model group showed a marked downregulation of the tight junction proteins ZO‐1 and occludin (Figure 6J–L). Til and FMT treatment robustly restored the expression of these proteins, whereas the A and AT groups failed to show significant improvement, indicating that antibiotic‐induced dysbiosis prevented the restoration of barrier integrity.

Consequently, the compromised barrier in the Model and antibiotic‐treated groups led to a significant surge in plasma LPS levels (Figure 6I), a condition known as metabolic endotoxemia. Til treatment effectively reduced plasma LPS concentrations, aligning with its ability to repair the gut barrier.

### 2.6 Til Reduced the Levels of TMAO Both In Vivo and In Vitro

In animal studies, Til treatment significantly reduced TMA and TMAO levels in both plasma and feces of ApoE^−/−^ mice, suggesting that Til remodels the gut microbiota to attenuate TMA/TMAO production. However, FMT from Til‐treated mice did not significantly reduce fecal TMA or TMAO levels, implying that the beneficial effects of Til may not be fully transferable through microbiota alone. These findings raised the possibility that Til or its metabolites may directly inhibit microbial TMA and TMAO production.

To further investigate this hypothesis, we first monitored plasma TMA and TMAO levels following a single oral gavage of Til. As shown in Figure S4, plasma TMA levels exhibited a downward trend over 24 h, but the changes were not statistically significant. In contrast, plasma TMAO levels were significantly reduced relative to the pre‐dose baseline at 8, 12, and 24 h post‐administration, with no significant change at 6 h post‐dose, the earliest time point sampled. These results indicate that Til can rapidly suppress systemic TMAO levels following oral administration.

To distinguish whether this effect was mediated primarily through systemic exposure or local gut action, a comparative study was performed via oral gavage and intraperitoneal (i.p.) injection (Figure 7A, B). Plasma and fecal concentrations of Til and its metabolites, along with TMA and TMAO levels, were measured. The i.p. injection group showed higher plasma concentrations of Til, Aca, and Aca‐7‐G but lower fecal concentrations compared to the oral group. Notably, Aca accumulated at the highest levels in fecal samples. No significant differences in plasma or fecal TMA levels were observed between the two administration routes. However, fecal TMAO levels were significantly reduced by 48% at 12 h and 36% at 24 h after oral administration, and this reduction was markedly greater than that seen with i.p. injection. Plasma TMAO levels did not differ significantly between the two administration routes. These results suggest that Til primarily acts locally in the gut to inhibit TMAO production, rather than through systemic circulation.

**FIGURE 7:**
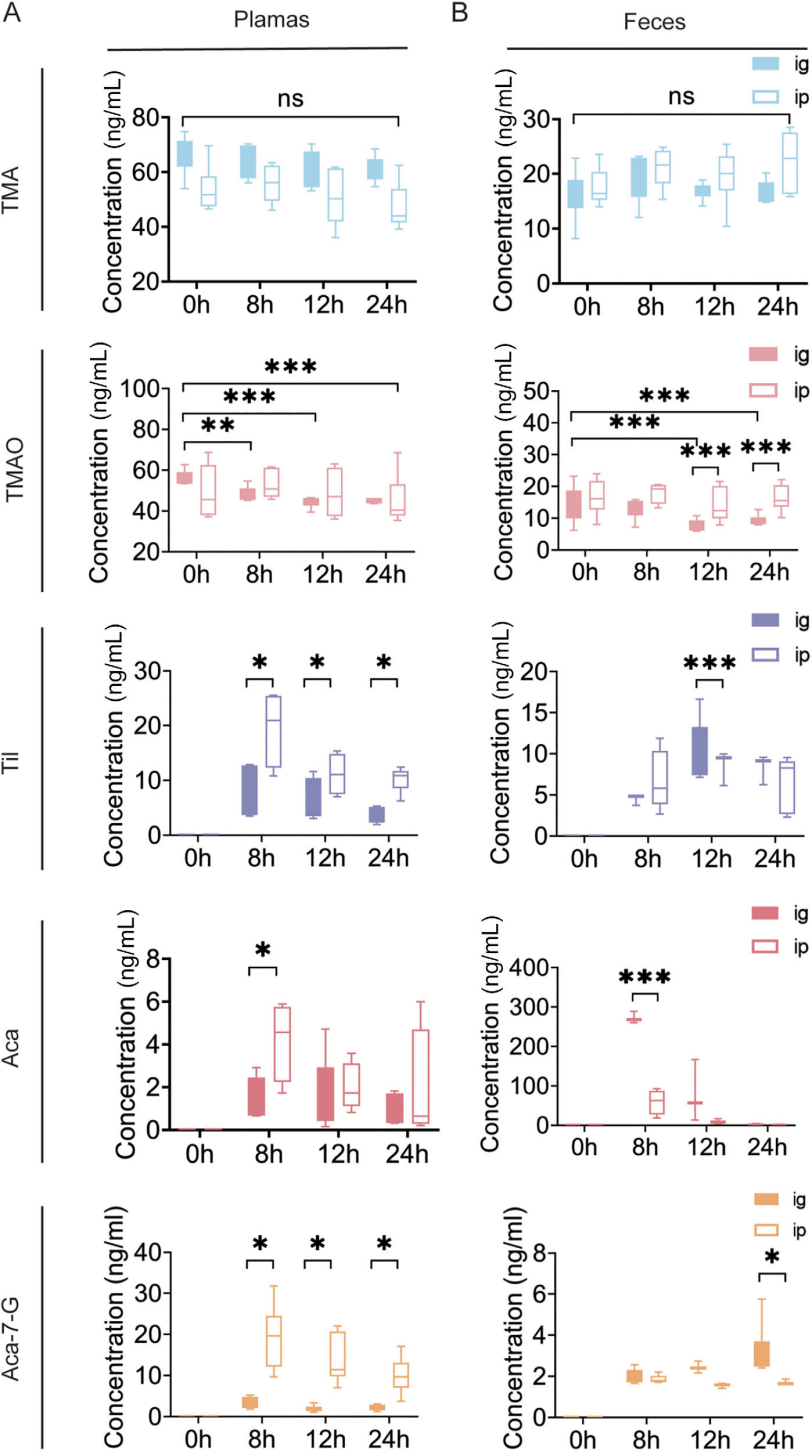
Acute oral gavage of Til directly reduced TMAO levels in both plasma and feces of mice. (A) Plasma levels of TMA, TMAO, Til, Aca, and Aca‐7‐G after oral and intraperitoneal administration. (B) Fecal levels of the above analytes following both administration routes. Data are shown as mean ± SD (*n* = 6). ns, not significant; ^*^ *p* < 0.05; ^**^ *p* < 0.01; ^***^ *p* < 0.001.

Since Aca was found at relatively higher concentrations in fecal samples, we hypothesized that it may contribute substantially to the observed effects. To test this, in vitro incubation assays were conducted using mice gut microbiota and liver homogenates, separately incubated with Til, Aca, and Aca‐7‐G. As shown in Figure 8A–C, none of the compounds significantly affected TMA or TMAO levels in the liver homogenate system, indicating minimal hepatic inhibition. In contrast, in the gut microbiota incubation system, all three compounds significantly suppressed TMAO production, with Aca demonstrating the strongest inhibitory effect. Moreover, residual compound analysis revealed that Aca remained the predominant species even when Til was the initial substrate, and that the inhibitory effect of Til was greater than that of Aca alone at 8 h, suggesting potential synergistic interactions.

**FIGURE 8:**
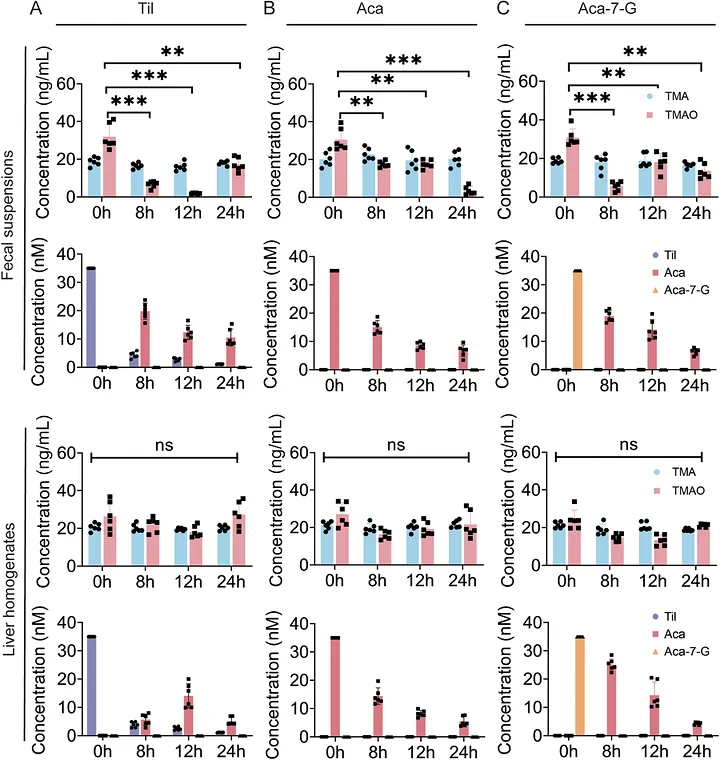
In vitro inhibition of TMAO production by Til and its metabolites. (A) Levels of TMA, TMAO, Til, Aca, and Aca‐7‐G following incubation of Til with fecal suspensions and liver homogenates. (B) Levels of TMA, TMAO, Til, Aca, and Aca‐7‐G following incubation of Aca with fecal suspensions and liver homogenates. (C) Levels of TMA, TMAO, Til, Aca, and Aca‐7‐G following incubation of Aca‐7‐G with fecal suspensions and liver homogenates. Data are shown as mean ± SD (*n* = 6). ^**^ *p* < 0.01; ^***^ *p* < 0.001; ns, not significant versus Control.

### 2.7 Til Directly Inhibits Microbial TMA‐to‐TMAO Conversion by Targeting TMM Enzyme

To identify the specific molecular targets of Til and its metabolites in the TMA metabolism pathway, we performed molecular docking and in vitro whole‐cell inhibitory assays.

Given our in vivo observation that Til significantly reduced TMAO without a concomitant decrease in TMA, we focused on the conversion step mediated by microbial enzymes. Molecular docking was employed to evaluate the binding affinities of Til, Aca, and Aca‐7‐G toward microbial TMM (a key enzyme for TMA oxidation), microbial CutC (the primary TMA‐lyase), and host hepatic FMO3 (Table S3). The results revealed that Til exhibited a robust binding affinity for microbial TMM (−9.3 ± 0.31 kcal/mol), which was notably stronger than its affinity for CutC (−6.8 ± 0.67 kcal/mol) (Table S3). Structural analysis showed that Til fits snugly into the active pocket of TMM, forming stable hydrogen bonds with key residues such as ASN‐72 and SER‐207 (Figure 9A, B). Although Aca‐7‐G showed a higher theoretical binding energy (−9.60 kcal/mol), the following functional assays highlighted the superior potency of the parent drug Til in a living microbial system.

**FIGURE 9:**
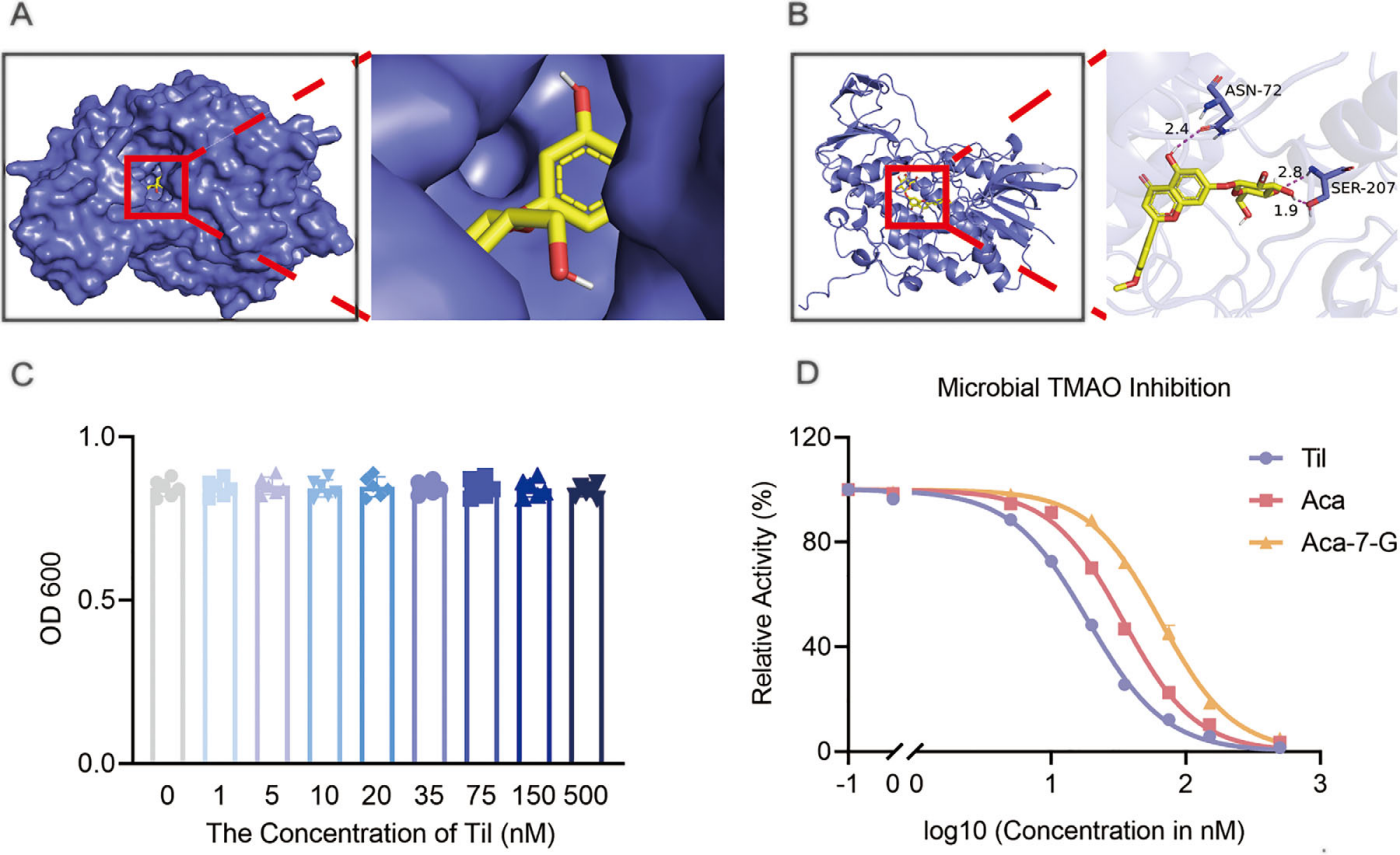
Til directly inhibits microbial TMA‐to‐TMAO conversion by targeting TMM enzyme. (A) Surface representation of the binding pocket of microbial TMM (AlphaFold model AF‐B8EIZ7‐F1, v4; UniProt B8EIZ7) with Til. (B) Detailed molecular interactions between Til and the active site of TMM; dashed lines indicate hydrogen bonds. (C) Bacterial growth (OD_600_) after 24‐h incubation with Til, Aca, and Aca‐7‐G (500 nM), showing no bactericidal effects. (D) Dose–response inhibition curves of Til, Aca, and Aca‐7‐G against TMAO production in *Aminobacter aminovorans*; curves are four‐parameter logistic fits to all individual replicate values. Data are presented as mean ± SD (*n* = 6). Binding affinities are given in Table S3 and IC_50_ values in Table S4.

To validate these findings, we conducted a whole‐cell inhibitory assay using *Aminobacter aminovorans*, a bacterial model with high TMM activity. First, we monitored the bacterial growth (OD_600_) and confirmed that Til, Aca, and Aca‐7‐G at concentrations up to 500 nM did not inhibit microbial growth (Figure 9C), indicating that the reduction in TMAO was due to enzymatic inhibition rather than bactericidal activity. Dose–response analysis demonstrated that Til is a highly potent inhibitor of microbial TMAO production, with an IC_50_ of 18.78 nM (95% CI: 18.40–19.17 nM), significantly outperforming Aca (IC_50_ = 34.22 nM) and Aca‐7‐G (IC_50_ = 64.72 nM) (Figure 9D and Table S4). Detailed concentration‐gradient data showed that Til inhibited TMAO generation in a clear dose‐dependent manner (Table S5).

Crucially, our pharmacokinetic data revealed that the peak fecal concentration of Til after oral administration reached approximately 35 nM. At this physiologically relevant concentration, Til achieved over 70% inhibition of TMAO production in vitro (relative activity: 25.6 ± 0.9%, Table S5). The close correspondence between the in vitro IC_50_ (18.78 nM) and the in vivo intestinal exposure (approximately 35 nM, i.e., within twofold of the IC_50_) indicates that the parent drug Til, rather than its metabolites, acts as the primary functional entity within the gut lumen to block the microbial conversion of TMA to TMAO.

## 3 Discussion

Til, a predominant flavonoid glycoside derived from medicinal‐edible plants such as *D. moldavica* L., has garnered increasing attention for its pleiotropic cardiovascular benefits. While its anti‐inflammatory and antioxidant properties are well‐documented, the precise role of the gut microbiota in mediating its anti‐atherosclerotic efficacy has remained elusive. In the present study, we demonstrated that Til exerts a potent, dose‐dependent protective effect on the vasculature in ApoE^−/−^ mice, significantly reducing plaque burden and enhancing plaque stability. Crucially, we identified that these therapeutic outcomes are not merely a result of systemic drug exposure but are fundamentally governed by a sophisticated “microbiota–metabolite–barrier” regulatory network.

A striking finding of our study is the “antibiotic paradox”: the depletion of gut microbiota via broad‐spectrum antibiotics (ABX) achieved the most profound reduction in pro‐atherogenic TMAO levels and significantly increased the systemic bioavailability of Til, yet the drug's benefit was only partly retained, and the dissociation was indicator‐specific. The gain in plaque stability was lost: collagen content in the AT group was significantly lower than in the Til group (*p* = 0.001) and no longer differed from the model group. By contrast, the reduction in overall plaque burden was preserved, lesion area in the AT group being indistinguishable from that in the Til group (*p* = 0.999), consistent with the microbiota‐independent anti‐inflammatory and antioxidant activities of Til demonstrated in our macrophage assays. Inflammatory cytokines in the A and AT groups did not differ significantly from the untreated model group. The lipid‐lowering arm, by contrast, was largely preserved under antibiotic co‐treatment: the AT group retained significant improvements in TG and HDL‐C, although its HDL‐C remained significantly below that of the Til group. We therefore propose that the microbiota gates the plaque‐stabilizing arm of Til's action rather than its activity as a whole. This PK–PD disconnect underscores that Til does not function as a traditional systemic agent whose efficacy is proportional to plasma concentration. Indeed, emerging evidence suggests that prolonged antibiotic use can actively promote plaque accumulation, primarily by triggering a cascade of secondary inflammatory insults that supersede the benefits of lowering TMAO. It should be noted, however, that neither antibiotic group showed aggravated AS relative to the model group on any index measured in the present study [37, 38]. Instead, our integrated profiling of the intestinal environment revealed that ABX‐induced dysbiosis creates a “metabolic and inflammatory void.” The near‐complete depletion of beneficial SCFAs, particularly butyrate, and the failure to restore intestinal barrier proteins (ZO‐1 and occludin remained at Model‐group levels) triggered severe metabolic endotoxemia. The resulting surge in plasma LPS likely drove a systemic inflammatory response that far outweighed the benefits of TMAO reduction. Crucially, this phenomenon is not restricted to antibiotic‐induced models. As highlighted by Getz and Reardon [39], previous research utilizing non‐antibiotic methods—such as germ‐free mouse models [40]—has consistently demonstrated that the total absence of microbiota can paradoxically accelerate plaque formation. This suggests that certain microbial components or metabolites are essential for maintaining immune homeostasis and vascular protection, and their loss (via either GF conditions or antibiotic depletion) outweighs the benefits of TMAO reduction. This highlights that a functional and balanced microbiota is a prerequisite for Til's therapeutic action, serving as a biological “processing plant” and a protective shield for the host.

The structural remodeling of the microbiota by Til, characterizedα by increased α‐diversity and the enrichment of *A. muciniphila*, appears to be a cornerstone of its chronic efficacy. *Akkermansia*, a well‐recognized probiotic, is known to strengthen the gut barrier by inducing the expression of tight junction proteins and modulating the mucus layer [41, 42]. In our study, the Til‐modulated microbiota (confirmed via FMT) not only reduced TMAO but also significantly restored SCFA levels. SCFAs, especially butyrate, serve as primary energy sources for colonocytes and act as signaling molecules that inhibit vascular inflammation via G protein‐coupled receptors (GPCRs) and histone deacetylase (HDAC) inhibition [43, 44]. The partial rescue of the anti‐atherosclerotic phenotype observed in FMT recipients, compared to the superior efficacy of direct Til administration, suggests a synergistic interaction: Til provides direct antioxidant protection to the gut epithelium, while the remodeled microbiota provides a sustained supply of protective metabolites.

Pharmacokinetic analysis in PGF mice further clarified the biotransformation pathway of Til. The significant increase in Til and its conjugate Aca‐7‐G in PGF mice, accompanied by a decrease in the aglycone Aca, indicates that gut microbiota‐derived enzymes (specifically β‐glucosidases) are essential for the β‐glycosidic hydrolysis of Til in the gut lumen [21]. However, the loss of efficacy in PGF mice, despite high Til levels, points to a more nuanced mechanism than simple metabolic activation. Our FMT data provided a critical clue: while plasma TMA/TMAO levels were reduced in recipients, fecal levels remained relatively stable. This suggests that a major component of the microbiota‐mediated effect is the reinforcement of the intestinal barrier, which limits the paracellular translocation of TMA from the gut lumen into the portal circulation, thereby depriving the liver of the substrate for TMAO synthesis [45].

Beyond structural remodeling, we pinpointed a direct, high‐affinity inhibitory mechanism against microbial TMAO biosynthesis. By utilizing molecular docking and in vitro whole‐cell functional assays, we identified microbial TMM—a key enzyme responsible for the flavin‐dependent oxidation of TMA to TMAO [46, 47]—as a primary target of Til. For these functional assays, we employed *A. aminovorans* (formerly *Pseudomonas aminovorans*), a well‐characterized model organism for methylotrophic metabolism with robust TMM activity, to accurately simulate the microbial conversion process in a living system [48, 49]. Remarkably, Til exhibited nanomolar inhibitory potency, which perfectly aligns with the peak fecal concentrations (∼35 nM) measured after oral dosing. This finding resolves the ambiguity regarding the active entity of Til. While its metabolite Aca also exhibits inhibitory potential in vitro, its functional contribution in vivo is likely constrained by rapid and extensive metabolic transformation. Our pharmacokinetic data, consistent with established models of flavonoid metabolism, indicate that Aca undergoes rapid Phase II metabolism (e.g., glucuronidation and sulfation) within the intestinal mucosa and liver [21]. This high metabolic clearance results in transient and limited exposure of the bioactive prototype Aca in both the gut lumen and systemic circulation. In contrast, the parent glycoside Til appears to be a more stable functional entity in the local intestinal environment, allowing for a more sustained and effective interaction with microbial TMM enzymes [50]. Moreover, the glycosylated structure of Til likely ensures superior solubility in the gut lumen and may facilitate active uptake by bacteria via carbohydrate transporters, allowing it to reach and inhibit intracellular TMM more effectively at low concentrations.

Furthermore, our comparative study of oral (intragastric, i.g.) versus intraperitoneal (i.p.) administration supports a site‐specific action of Til. Despite higher systemic exposure, i.p. administration resulted in lower fecal concentrations of Til and its metabolites, and a markedly smaller reduction in fecal TMAO than oral administration. These results argue against inhibition of host hepatic FMO3 as the primary driver in vivo. Instead, Til likely needs to be present locally in the gut lumen to interact with the microbiota. This “gut‐local” mechanism may be advantageous for long‐term AS therapy, as it could minimize the risk of systemic toxicity and off‐target effects associated with high‐dose systemic FMO3 inhibitors, which can lead to trimethylaminuria (fish‐odor syndrome) and hepatic strain [51, 52].

It is important to note that Til possesses intrinsic anti‐inflammatory and antioxidant activities that are, in part, independent of the gut microbiota, as demonstrated by our in vitro macrophage assays. However, our in vivo antibiotic‐depletion data indicate that the full therapeutic expression of these pleiotropic activities—including lipid‐lowering, cytokine modulation, and plaque stabilization—is contingent upon a functional gut microbiota. We therefore propose that the gut microbiota–metabolite–barrier axis functions as a permissive upstream determinant that gates the in vivo efficacy of Til's multiple pharmacological activities, rather than operating as an isolated, independent mechanism. One limitation should be acknowledged: serum cytokines could be measured in only four animals per group, so these comparisons were underpowered. The reductions in TNF‐α and IL‐1β and the rise in IL‐10 were of a magnitude comparable to the significant fall in IL‐6 (Hedges’ g = 1.5–2.0) but did not reach significance, and the anti‐inflammatory activity of Til is therefore supported here principally by the macrophage polarization and ROS experiments.

In conclusion, our study establishes that the anti‐atherosclerotic efficacy of Til is mediated by a dual microbiota‐dependent mechanism: the structural restoration of microbial homeostasis (boosting SCFAs and barrier integrity) and the direct functional inhibition of microbial TMM activity. By maintaining the “gut microbiota–metabolite–barrier” axis, Til achieves a level of vascular protection comparable to clinical statins, while offering a unique multi‐pathway regulatory advantage. These findings provide a compelling rationale for the development of Til as a microbiota‐targeted therapy and offer a new conceptual framework for evaluating the therapeutic potential of other dietary flavonoids in cardiovascular medicine.

## 4 Conclusion

In conclusion, our study demonstrates that the anti‐atherosclerotic efficacy of Til critically depends on the presence of a functional gut microbiota. Til and its metabolites engage in bidirectional interactions with the intestinal ecosystem, reciprocally restructuring the microbial ecology while undergoing metabolic activation. Crucially, Til's therapeutic outcomes are mediated by a multi‐faceted microbiota–metabolite–barrier axis, which involves the direct inhibition of microbial TMM activity at nanomolar concentrations, the restoration of beneficial SCFAs, and the reinforcement of intestinal barrier integrity. These integrated pathways collectively suppress systemic inflammation and metabolic endotoxemia. Our findings establish a refined conceptual framework for flavonoid‐based drug development, highlighting that the pivotal engagement of functional microbiota is a prerequisite for achieving optimal multi‐target therapeutic efficacy in CVDs.

## 5 Materials and Methods

### 5.1 Chemicals and Materials

Til, Aca, and Aca‐7‐G reference standards were purchased from Chengdu Yirui Biotechnology Co. Ltd. (Chengdu, China). Mass spectrometry grade formic acid and acetonitrile were obtained from Merck KGaA (Darmstadt, Germany). Suberoylanilide hydroxamic acid (SAHA, > 98%) was from Shanghai Yuanye Biotechnology Co. Ltd. (Shanghai, China). Antibiotics (neomycin, ampicillin, metronidazole, and vancomycin) were sourced from Chengdu Tongde Pharmaceutical Co. Ltd. (Chengdu, China). Enzyme‐linked immunosorbent assay (ELISA) kits for mouse TNF‐α, IL‐1β, IL‐6, and IL‐10 were purchased from Shanghai Kehansheng Biotechnology Co. Ltd. (Shanghai, China), and TMA/TMAO detection kits were from Shanghai Yuanju Biotechnology Center (Shanghai, China).

### 5.2 Experimental Animals

Specific pathogen‐free grade male C57BL/6J mice and ApoE^−/−^ mice (6 weeks old, 20–22 g) were purchased from Beijing HFK Bioscience Co. Ltd. (Beijing, China). All mice were housed individually under controlled conditions: temperature 20°C–25°C, humidity 30%–50%, and a 12‐h light/dark cycle. Animals had free access to food and water, with bedding and drinking water changed regularly. All procedures involving animals were reviewed and approved by the Ethics Committee of West China Hospital (approval no.: 220231213004).

### 5.3 Construction of the AS Mouse Model

A stable AS model was established in ApoE^−/−^ mice by feeding a high‐fat diet, a widely accepted approach that closely mimics the progression of human AS. The high‐fat diet (formula no. H10540) was supplied by Beijing HFK Bioscience Co. Ltd. (Beijing, China). H10540, formulated with reference to Research Diets D12108C, is a high‐fat, high‐cholesterol diet in which fat provides 40% of total calories (sourced from cocoa butter and soybean oil), protein 20%, and carbohydrate 40% (total energy density 4.52 kcal/g), supplemented with 1.254% (w/w) cholesterol; full composition is provided in Table S12. To induce AS, ApoE^−/−^ mice (6–8 weeks old) were first acclimated on a standard diet for 1 week, followed by continuous feeding with the high‐fat diet for 12 weeks.

### 5.4 Dose–Response Pilot Study

To establish the optimal therapeutic dose of Til (Figure S1), ApoE^−/−^ mice with the AS model already established by the vendor were randomized into six groups: Control, Model, Til‐L (2.1 mg/kg/d), Til‐M (3.5 mg/kg/d), Til‐H (7 mg/kg/d), and Simvastatin (3.5 mg/kg/d), and received the corresponding oral treatment for 12 weeks. This pilot cohort was independent of, and shorter in total high‐fat diet duration than, the primary mechanistic cohort used in Figure 2.

### 5.5 Construction and Validation of the PGF Mouse Model

C57BL/6J mice were randomly divided into control and PGF groups, with ad libitum access to food and water. PGF mice received a daily oral gavage of neomycin, ampicillin, metronidazole (each 5 mg/kg), and vancomycin (2.5 mg/kg) for 5 consecutive days, followed by a 7‐day antibiotic washout period. PGF status was verified by culturing fecal samples on nutrient agar under anaerobic conditions on Day 5; significant reduction or absence of microbial colonies indicated successful gut microbiota depletion. This 5‐day course followed by a 7‐day washout was used only for the pharmacokinetic experiments; in the efficacy study, the antibiotic groups received the same combination daily for 12 weeks [53, 54].

### 5.6 Cell Culture

RAW264.7 murine macrophage cells were obtained from the American Type Culture Collection (ATCC). Cells were cultured in Dulbecco's Modified Eagle Medium (DMEM) supplemented with 10% fetal bovine serum (FBS), 100 µg/mL penicillin, and 100 µg/mL streptomycin, at 37°C in a humidified incubator containing 5% CO_2_. Cells were subcultured using a cell scraper once they reached over 80% confluence.

### 5.7 Antibiotic‐Induced Gut Microbiota Depletion and FMT

*Experimental grouping*: C57BL/6J mice were used as the control group. ApoE^−/−^ AS mice were randomly assigned to five groups: model, antibiotic model (A), Til treatment (Til), antibiotic + Til treatment (AT), and FMT. The control group received normal saline, and the model group received the same. Group A received neomycin, ampicillin, metronidazole (each 5 mg/kg) and vancomycin (2.5 mg/kg) by daily oral gavage throughout the 12‐week treatment period. Group Til received 7 mg/kg Til daily. Group AT received both 7 mg/kg Til daily and the same antibiotic regimen as Group A, also for 12 weeks. In the FMT group, recipient mice first received the antibiotic regimen described above to deplete the resident microbiota. Fresh feces were then collected from Til‐treated donor mice, pooled in equal amounts, suspended in sterile PBS at 100 mg/mL, vortexed to homogeneity and passed through a 70 µm cell strainer. The suspension was kept on ice throughout and administered within 30 min of preparation; each recipient received 200 µL by oral gavage once daily for 12 weeks. All AS model mice were fed a high‐fat diet for 12 weeks to establish the AS model, followed by an additional 12 weeks of concurrent drug treatment with continued high‐fat diet feeding, for a total high‐fat diet exposure of 24 weeks (Figure 1A).

### 5.8 Gut Microbiota Analysis by 16S rRNA Sequencing

After the final gavage, mice were fasted for 12 h with free access to water. Fresh fecal samples were collected under sterile conditions, frozen in liquid nitrogen, and stored at −80°C. Genomic DNA was extracted, and its purity and concentration were assessed. DNA was diluted to 1 ng/µL, PCR amplification was performed, and products were verified by electrophoresis. Libraries were constructed, validated, and subjected to high‐throughput sequencing.

### 5.9 Gross Aortic ORO Staining

Aortas fixed in 4% paraformaldehyde were rinsed with saline, and adipose tissue was removed under a stereomicroscope. Aortas were incubated with ORO solution, differentiated with 75% ethanol, rinsed with saline, and spread flat on a black background for photography. Stained areas were analyzed using ImageJ software.

### 5.10 Histological Analysis of Aortic Valves Using H&E, Masson, and ORO Staining

*Frozen section preparation*: The heart apex was removed and the tissue embedded in OCT compound. Continuous 5‐µm sections were cut using a cryostat at −20°C.

*H&E staining*: Sections were stained with hematoxylin for 5–10 min, rinsed with water, differentiated with 1% hydrochloric acid ethanol, stained with eosin for 3–5 min, and dehydrated through a graded ethanol series. After clearing in xylene, sections were mounted with neutral balsam.

*Masson's trichrome staining*: Sections were hydrated through an ethanol gradient, stained with Weigert's iron hematoxylin for 5–10 min, followed by acid fuchsin for 5 min and aniline blue for 5–10 min. Sections were dehydrated, cleared, and mounted as above.

*ORO staining*: Sections were hydrated, immersed in 0.3%–0.5% ORO solution for 10–15 min, rinsed with 70% isopropanol or water and then dehydrated, cleared, and mounted with transparent medium.

Stained areas were analyzed using ImageJ software. Necrotic core areas were defined as acellular, matrix‐poor regions within the plaque, frequently containing cholesterol clefts, identified on H&E‐stained sections; areas were manually outlined and expressed as a percentage of total plaque area.

### 5.11 Detection of Blood Lipid Levels

Blood samples from each mouse were collected, allowed to stand, and then centrifuged at 8000 rpm for 10 min at 4°C to obtain the serum. Blood lipid profiles, including TC, TG, LDL‐C, and HDL‐C, were determined using a biochemical analyzer.

### 5.12 Detection of Inflammatory Factor and TMA/TMAO Levels

*ELISA for inflammatory cytokines and TMA/TMAO levels*: Mouse serum samples were analyzed using a double‐antibody one‐step sandwich ELISA. Pre‐coated micro‐wells received samples, standards, and HRP‐conjugated detection antibody. After incubation at 37°C and washing, TMB substrate was added, and color development was allowed. Absorbance at 450 nm was measured within 15 min using a microplate reader. Concentrations of TNF‐α, IL‐1β, IL‐6, IL‐10, TMA, and TMAO were calculated from the optical density values. Owing to the limited volume of serum obtainable from each animal, the full four‐cytokine panel could be completed for only four animals per group, whereas all other endpoints were measured in six animals per group. The four animals were those for which sufficient serum remained; no sample was excluded on the basis of its measured value.

### 5.13 UPLC‐MS/MS Conditions

The concentrations of Til and its metabolites were measured using an LC‐20AXR UHPLC system (Shimadzu, Kyoto, Japan) coupled with a QTRAP 5500 triple quadrupole mass spectrometer (AB Sciex, Framingham, MA, USA). Chromatographic separation was performed on a Waters Acquity UPLC BEH C18 column (1.7 µm, 2.1 × 100 mm) with a mobile phase of 0.01% formic acid in water (A) and acetonitrile (B), using gradient elution at 0.5 mL/min. The column was maintained at 35°C, and the injection volume was 5 µL. The gradient was: 0–0.01 min, 0% → 10% B; 0.01–2.5 min, 10% → 90% B. The autosampler was set at 4°C.

Mass detection employed a positive electrospray ionization (ESI) source in multiple reaction monitoring (MRM) mode. Parameters were: ion spray voltage, 5500 V; declustering potential, 120 V; source temperature, 580°C; curtain gas, 30 psi; GS1, 55 psi; GS2, 60 psi. MRM transitions were: Til, *m/z* 447.1→270.1 (CE: 58 V); Aca, 285.1→242.1 (CE: 45 V); Aca‐7‐G, 285.1 → 242.1 (CE: 61 V); SAHA (IS), 265.2 → 232.2 (CE: 18 V).

### 5.14 Quantification of Til in Plasma From PGF Mice

After successful model establishment, control and PGF mice were randomly divided into low‐dose (7 mg/kg) and high‐dose (21 mg/kg) i.g. administration groups based on our previous studies. Blood samples (20–35 µL) were collected via retro‐orbital venous plexus at baseline and at 5, 15, 30 min, 1, 3, 5, 8, 12, and 24 h post‐administration using heparinized capillaries. Plasma was obtained by centrifugation (8000 rpm, 10 min) and stored at −80°C. For analysis, 10 µL of plasma was mixed with 60 µL acetonitrile containing SAHA (20 ng/mL) as internal standard. After vortexing (1 min) and centrifugation (13,500 rpm, 15 min), 50 µL of the supernatant was subjected to UPLC‐MS/MS.

### 5.15 Macrophage Polarization Assay

Blank control wells were maintained under normal culture conditions. The remaining cells were stimulated with LPS (100 ng/mL) and interferon‐gamma (IFN‐γ, 20 ng/mL), and incubated for 24 h to induce M1 macrophage polarization. Following stimulation, the medium was removed and replaced with fresh medium containing Til, Aca, or Aca‐7‐G (final concentration: 10 µmol/L). Cells were incubated for an additional 12 h. After incubation, cells were collected, stained as required, and prepared for flow cytometric analysis.

### 5.16 Antioxidant Assay

*Flow cytometry for ROS detection*: Cells (3 × 10^5^ cells/mL) were seeded in 24‐well plates, induced with a ROS reagent for 40 min, and treated with Til, Aca, or Aca‐7‐G (10 µmol/L) for 12 h. After PBS washing, cells were stained with DCFH‐DA for 40 min, washed again, and analyzed by flow cytometry.

*Confocal microscopy for ROS imaging*: Cells (2 × 10^5^ cells/mL) were seeded in confocal dishes, treated and stained as above. After PBS washing, fluorescence images were captured using laser confocal microscopy and analyzed with ZEN 2012.

### 5.17 In Vivo Experiments on TMA/TMAO Inhibition by Til and Its Metabolites

*Animal grouping and sample collection*: Twelve AS model mice were randomly divided into two groups: oral Til (7 mg/kg, gavage) and i.p. Til (7 mg/kg, i.p.). Blood samples were collected via retro‐orbital venous plexus using heparinized capillaries at 0 (pre‐dose), 8, 12, and 24 h post‐administration (20–35 µL per time point). Samples were centrifuged at 8000 rpm for 10 min to obtain plasma, then stored at −80°C. After dosing, mice were housed in metabolic cages for fecal collection at corresponding time points. Feces were mixed with sterile anaerobic broth (1:10, m:v), centrifuged, and supernatants stored at −80°C.

### 5.18 In Vitro Incubation Assays on TMA/TMAO Inhibition by Til and Its Metabolites

Fresh feces from AS model mice were mixed with sterile anaerobic broth at a 1:30 (m:v) ratio, filtered, and saturated with N_2_. The bacterial suspension was pre‐incubated at 37°C for 30 min. Til, Aca, and Aca‐7‐G were added to achieve a final concentration of 35 nM. Anaerobic conditions were maintained by continuous N_2_ flushing. The incubation system was cultured at 37°C, 200 rpm for 8, 12, and 24 h. Samples were collected at each time point and stored at −80°C for further analysis.

### 5.19 Quantification of SCFAs

The concentrations of SCFAs (acetate, propionate, butyrate, isobutyrate, valerate, isovalerate, and hexanoate) were determined using an LC‐MS/MS‐based derivatization method as previously described with modifications. Briefly, samples were extracted with acetonitrile and derivatized using 3‐nitrophenylhydrazine (3‐NPH) and 1‐ethyl‐3‐(3‐dimethylaminopropyl) carbodiimide (EDC) in a pyridine‐buffered system at 30°C for 30 min. The derivatized analytes were separated on a Waters ACQUITY UPLC BEH C18 column (1.7 µm, 2.1 mm × 100 mm) using an LC‐30A UHPLC system (Shimadzu) and detected on a QTRAP 4000 mass spectrometer (AB Sciex) operating in negative ESI and MRM modes. Data were normalized to sample weight or volume. Regression equations and linear ranges for the SCFA analytes are given in Table S10, and representative chromatograms in Figure S7. Detailed instrumental parameters and gradient profiles are provided in Supporting Information.

### 5.20 Detection of Plasma LPS (Endotoxemia)

Systemic endotoxin levels (LPS) in mouse plasma were measured using a chromogenic limulus amebocyte lysate (LAL) assay kit following the manufacturer's instructions. To avoid interference, plasma samples were diluted and pre‐heated at 70°C for 10 min to inactivate plasma inhibitors. All materials used for blood collection and processing were endotoxin‐free. The absorbance was measured at 405 nm, and the concentration was calculated against an *E. coli* endotoxin standard curve, expressed as endotoxin units per milliliter (EU/mL).

### 5.21 Western Blotting for Intestinal Tight Junction Proteins

Total proteins were extracted from mouse ileal tissues using RIPA lysis buffer supplemented with protease and phosphatase inhibitors. Protein concentrations were determined using a BCA protein assay kit. Equal amounts of protein (30 µg) were resolved by SDS‐PAGE. Specifically, 6% gels were used for the high‐molecular‐weight protein ZO‐1 (approx. 220 kDa), while 10% gels were used for occludin (approx. 65 kDa). Proteins were then transferred onto PVDF membranes using a wet transfer system (constant 300 mA for 150 min for ZO‐1; 90 min for occludin). The membranes were blocked with 5% non‐fat milk in TBST for 1 h at room temperature and then incubated overnight at 4°C with primary antibodies against ZO‐1 (1:1000), occludin (1:1000), and GAPDH (1:5000) as an internal control. After washing with TBST, the membranes were incubated with HRP‐conjugated secondary antibodies (1:5000) for 1 h. Protein bands were visualized using an enhanced chemiluminescence (ECL) detection system and quantified by densitometry using ImageJ software. All target protein levels were normalized to their respective GAPDH levels.

### 5.22 In Vitro Whole‐Cell Microbial Inhibition Assay

To validate the direct inhibitory effects of Til and its metabolites on microbial TMAO biosynthesis, a whole‐cell inhibitory assay was performed using *A. aminovorans* (ATCC 15148), a bacterial strain characterized by robust TMM activity.

*Assay conditions and treatment*: Bacteria were cultured in a minimal salts medium (MSM) at 30°C until the logarithmic growth phase. The bacterial suspension was then harvested and adjusted to a final optical density (OD_600_) of 0.5 using sterile MSM. TMA hydrochloride (TMA·HCl) was added as the substrate at a final concentration of 100 µg/mL. To determine the half‐maximal inhibitory concentration (IC_50_), Til, Aca, and Aca‐7‐G were added to the culture at a series of concentration gradients (0, 1, 5, 10, 20, 35, 75, 150, and 500 nM). Notably, the 35 nM concentration point was specifically included to match the peak physiological fecal concentration of Til measured in our in vivo pharmacokinetic study. Each concentration group was tested in six independent biological replicates (*n* = 6).

*Incubation and sample preparation*: The reaction mixtures were incubated anaerobically in a shaking incubator (150 rpm) at 30°C for 24 h. At the end of the incubation, OD_600_ was measured for each sample to assess bacterial viability and exclude potential bactericidal effects. The reaction was terminated by adding an equal volume of ice‐cold acetonitrile (containing the internal standard). After vortexing and centrifugation (12,000 rpm, 10 min at 4°C), the supernatants were collected for metabolite analysis.

*Quantification and statistics*: The concentrations of TMA and TMAO were determined using a validated LC‐MS/MS method (regression equations and linear ranges, Table S11; representative total ion chromatograms, Figure S6). Relative enzyme activity was calculated for each biological replicate as the percentage of TMAO produced relative to the 0 nM control of that same replicate, and is reported as mean ± SD of the six replicates. IC_50_ values were obtained with a four‐parameter logistic nonlinear regression model (log[inhibitor] versus normalized response, with the upper and lower asymptotes constrained to 100% and 0%) in GraphPad Prism 7.0. The model was fitted to all individual replicate values—nine concentrations × six biological replicates, giving 54 points per compound—rather than to the concentration means, and the 95% confidence intervals reported in Table S4 are those returned by that fit.

### 5.23 Statistical Analysis

The concentration–time curve was constructed using GraphPad Prism 7.0. Pharmacokinetic parameters were calculated using DAS software (Version 3.2.6). Statistical analysis was performed with SPSS 20.0. Normality was assessed by the Shapiro–Wilk test, and homogeneity of variance by Levene's test. Normally distributed data with equal variances were compared by one‐way ANOVA. Because each treatment group was compared against a single reference group, comparisons against the model group were made with Dunnett's multiple‐comparison test; the pre‐specified contrast between the AT and Til groups was evaluated by Tukey's HSD. Where variances were unequal, Welch's ANOVA with Tamhane's T2 post hoc test was applied. Data are presented as mean ± SD. A significance level of *α* = 0.05 was used (two‐tailed), and “ns” denotes *p* > 0.05. Unless stated otherwise, comparisons are made against the model group.

## Author Contributions

X.R., R.L., L.W., and W.C. revised and drafted the manuscript, and contributed to conceptualization, methodology, data curation, and formal analysis; L.W. also contributed to funding acquisition. M.G., Z.H., Y.P.Z., and Y.L. contributed to methodology, data curation, and formal analysis. Y.G. performed data curation. C.H., Y.LZ., and Y.Y. handled project administration. X.S. and X.W. contributed to conceptualization, formal analysis, funding acquisition, and supervision. X.R., R.L., W.C., and L.W. contributed equally to this work. All authors have read and agreed to the published version of the manuscript.

## Funding

This work was supported by the Tianshan Talent Program: Leading Talent in Medicine and Health (grant number TSYC202401A016) and the Science and Technology Project of Xinjiang Production and Construction Corps (grant numbers 2022AB020, 2023AB045, and 2024AB071), awarded to Xinchun Wang, and the Tianchi Academic Young PhD Program, awarded to Liping Wang. The funders had no role in the study design; data collection, analysis, or interpretation; manuscript preparation; or the decision to submit the manuscript for publication.

## Ethics Statement

All animal procedures were reviewed and approved by the Ethics Committee of West China Hospital, Sichuan University (approval number: 220231213004), and were performed in accordance with the institutional guidelines for the care and use of laboratory animals and with the ARRIVE guidelines.

Clinical experiments: Not applicable. This study did not involve human participants, human tissue or human data, and no clinical approval number is therefore reported.

## Conflicts of Interest

The authors declare no conflicts of interest.

## Supporting information




**Supporting File 1**: mco271141‐sup‐0001‐SuppMat.docx

## Data Availability

The raw sequence data reported in this paper have been deposited in the Genome Sequence Archive (Genomics, Proteomics & Bioinformatics 2025) in National Genomics Data Center (Nucleic Acids Res 2025), China National Center for Bioinformation/Beijing Institute of Genomics, Chinese Academy of Sciences (GSA: CRA052199) that are publicly accessible at https://ngdc.cncb.ac.cn/gsa. All other raw data supporting the findings of this study are available from the corresponding author upon reasonable request.
