# Supplementary material for: Dual Gut Microbiota‐Dependent Mechanisms Underlying the Anti‐Atherosclerotic Effect of Tilianin
Source: MedComm (2020). 2026 Nov 8;7(11):e71141. doi: 10.1002/mco2.71141 (PMC13744651; doi:10.1002/mco2.71141)
Supplement: Supplementary file 1 [file MCO2-7-e71141-s001.docx]

**Supplementary Materials for**

**Dual gut-microbiota-dependent mechanisms underlying the anti-atherosclerotic effect of tilianin**

**Author Names:**

Xuli Ruan ^a, b1^, Ruyue Liu ^a, b1^, Wenjiang Cao ^a1^, Liping Wang ^b1^, Mengran Guo c, Zhongshan He ^c^, Yupei Zhang ^d^, Yaoyao Luo ^c^, Yuntao Gao ^a, b^, Chuansheng Huang ^a^, Yunli Zhao ^a^, Yong Yuan ^a^, Xiangrong Song ^c*^ and Xinchun Wang ^a*^

**Author Affiliations:**

^a^ Department of Pharmacy, First Affiliated Hospital of Shihezi University, Shihezi 832008, China.

^b^ Key Laboratory of Xinjiang Phytomedicine Resource and Utilization, Ministry of Education, School of Pharmacy, Shihezi University, Shihezi 832002, China.

^c^ Department of Critical Care Medicine, Department of Clinical Pharmacy, State Key Laboratory of Biotherapy and Cancer Center, West China Hospital, Sichuan University, Chengdu 610000, China.

^d^ Department of Head and Neck Surgery, Sichuan Clinical Research Center for Cancer, Sichuan Cancer Hospital & Institute, Sichuan Cancer Center, University of Electronic Science and Technology of China, Chengdu, 610040, China

***Correspondence:** Xinchun Wang ([cwjwxc@163.com](mailto:cwjwxc@163.com)), Xiangrong Song (songxr@scu.edu.cn)

1 These authors contributed equally to this work.

This PDF file includes:

Supplementary Methods

Figures S1 to S7

Tables S1 to S12

**Supplementary Methods**

**Preparation of standard solutions**

Accurately weigh 1.0 mg each of Til, Aca, and Aca-7-G standards. Dissolve in DMSO to obtain 1 mg/mL standard stock solutions. Store at 4 °C until use.

**Preparation of oral solution**

Til oral solution: Accurately weigh 6.3 mg of Til powder into an EP tube and dissolve in 0.9% sodium chloride solution to a final volume of 1 mL (final concentration: 6.3 mg/mL; 1% DMSO).

Antibiotic suspension: PGF mice received a daily oral gavage of neomycin, ampicillin, metronidazole (each 5 mg/kg), and vancomycin (2.5 mg/kg)

**Preparation of acetonitrile solution containing SAHA (IS)**

Accurately weigh SAHA and dissolve in methanol to prepare a 1 mg/mL stock solution. Dilute an appropriate volume of this stock in acetonitrile to a final concentration of 20 ng/mL. Store at 4 °C until use.

**Sample pretreatment**

Add 60 μL of acetonitrile containing SAHA (20 ng/mL) to 10 μL of plasma. Vortex for 1 min and centrifuge at 13,500 rpm for 15 min. Collect 50 μL of the supernatant for UPLC-MS/MS analysis.

**Methodological Investigation**

**Exclusivity experiment**

The potential interference from endogenous plasma components was assessed by comparing chromatograms of blank plasma, blank plasma spiked with reference standards, and actual plasma samples. Representative chromatograms for Til, Aca, and Aca-7-G in C57BL/6J mouse plasma are shown in Figure S5. No interfering peaks were observed in blank plasma at the retention times of the analytes. The retention times for Til, Aca, and Aca-7-G were 1.54 min, 1.76 min, and 1.55 min, respectively, indicating that the method exhibits good specificity.

**Preparation of the standard curve**

To prepare calibration samples, 10 μL of blank plasma was mixed with 50 μL of acetonitrile containing SAHA (IS, 20 ng/mL) and 10 μL of mixed standard solution. Calibration concentrations for Aca-7-G were: 400, 200, 100, 50, 25, 12.5, 6.25, and 3.125 ng/mL. For Til and Aca, the concentrations were: 100, 50, 25, 12.5, 6.25, 3.125, 1.5625, and 0.7812 ng/mL. Samples were vortexed for 1 min and centrifuged at 13,500 rpm for 15 min. A 50 μL aliquot of the supernatant was then analyzed by UPLC-MS/MS.

The standard curves and limits of quantification are summarized in Table S6. The calibration curves for all target analytes in plasma showed good linearity, with correlation coefficients (r) exceeding 0.9.

**Precision and accuracy**

Blank plasma was used to prepare six QC plasma samples at high, medium, and low concentrations (prepared as described in the sample pretreatment section). The concentrations of each compound in the QC samples were determined using the corresponding standard curve on the same day. Intra-day precision and accuracy were calculated accordingly. The QC samples were analyzed over three consecutive days to assess inter-day (diurnal) precision and accuracy. Concentrations were determined using the respective standard curves generated each day. As shown in Table S7, both intra-day and inter-day precision for Til and its metabolites met the acceptance criteria.

**Stability**

Blank plasma was used to prepare three QC samples (high, medium, and low concentrations) to evaluate sample stability under different conditions: short-term stability (room temperature for 12 h), freeze–thaw stability (three cycles between −20 °C and room temperature), and long-term stability (stored at −20 °C for 14 days). As shown in Table S8, the RSDs of peak area ratios for all conditions were below 15%, indicating that the analytes were stable under the tested conditions.

**Recovery rate and matrix effect**

Extraction recovery and matrix effect were calculated as follows:

Recovery = A / B

Matrix effect = B / C

Sample pretreatment steps:

Pretreated plasma: Add 200 μL methanol to 25 μL blank plasma, vortex for 3 min, centrifuge at 14,000 rpm for 10 min, collect supernatant.

Pretreated water: Add 200 μL methanol to 25 μL ultrapure water, vortex for 3 min, centrifuge at 14,000 rpm for 10 min, collect supernatant.

Sample groups:

A: 10 μL blank plasma + 50 μL acetonitrile containing SAHA (IS, 20 ng/mL) + 10 μL QC solution → vortex 1 min → centrifuge 13,500 rpm, 15 min → 50 μL supernatant for UPLC-MS/MS.

B: 10 μL pretreated plasma + 50 μL acetonitrile containing SAHA (IS, 20 ng/mL) + 10 μL QC solution → same as above.

C: 10 μL pretreated water + 50 μL acetonitrile containing SAHA (IS, 20 ng/mL) + 10 μL QC solution → same as above.

Extraction recovery rates and matrix effects are presented in Table S9. Matrix effects at all QC levels ranged from 79.8% to 103.8%, indicating minimal interference. Recovery rates were high and consistent across compounds.

**Quantification of Fecal Short-Chain Fatty Acids (SCFAs) via Derivatization-Based LC-MS/MS**

**Instrumentation and Reagents**

Chromatographic separation and mass spectrometric detection were performed using a Shimadzu Nexera LC-30A UHPLC system coupled with an AB SCIEX QTRAP 4000 mass spectrometer. Additional equipment included an ultra-low temperature freezer (902-ULTS, Thermo Fisher Scientific), a Milli-Q Advantage A10 water purification system (Millipore), and a refrigerated high-speed centrifuge (5430R, Eppendorf). LC-MS grade methanol and acetonitrile were purchased from Honeywell. Ammonium acetate, 3-nitrophenylhydrazine hydrochloride (3-NPH·HCl), and 1-ethyl-3-(3-dimethylaminopropyl) carbodiimide hydrochloride (EDC·HCl) were obtained from Sigma-Aldrich.

**Sample Preparation and Derivatization**

(1) Fecal or serum samples (40 μL) were mixed with 200 μL of acetonitrile. The mixture was vortexed for 3 min and subjected to ultrasonication for 10 min to obtain a homogeneous suspension. After centrifugation at 14,000 × g and 4°C for 10 min, the supernatant was collected.
(2) For derivatization, 40 μL of the sample supernatant (or standard solution) was mixed with 20 μL of 200 mmol/L 3-NPH solution. Subsequently, 20 μL of 120 mmol/L EDC-6% pyridine solution was added. The reaction was conducted in a water bath at 30°C for 30 min, followed by rapid cooling for 1 min.
(3) The final volume was adjusted to 200 μL with 10% acetonitrile, vortexed, and centrifuged at 14,000 × g for 10 min. The resulting supernatant was used for LC-MS/MS analysis.
(4) Quality control (QC) samples were prepared by pooling equal aliquots from each test sample to monitor instrumental stability.

**LC-MS/MS Conditions**

Chromatographic separation was achieved on a Waters ACQUITY UPLC BEH C18 column (1.7 μm, 2.1 mm × 100 mm) at 40°C. The mobile phase consisted of 0.1% formic acid in water (A) and 0.1% formic acid in acetonitrile (B). The flow rate was 0.25 mL/min with an injection volume of 5 μL. The autosampler was maintained at 10°C. The gradient program was as follows: 0–1 min, 10% B; 1–2 min, 10–50% B; 2–5 min, 50–60% B; 5–6 min, 60–98% B; 6–7 min, 98% B; 7–7.01 min, 98–10% B; and 7.01–8 min, 10% B.

Mass spectrometry was performed in negative electrospray ionization (ESI−) mode. Parameters were set as follows: ion spray voltage: -4500 V; nebulizer gas (Gas1): 50 psi; heater gas (Gas2): 50 psi; curtain gas (CUR): 35 psi; source temperature: 550°C. Analytes were detected using Multiple Reaction Monitoring (MRM) mode.

**LC-MS/MS Quantification of TMA and TMAO in Enzyme Inhibition Assays**

**Instrumentation**

The analysis was performed using a high-performance analytical platform consisting of an ultra-low temperature freezer (902-ULTS, Thermo Fisher Scientific), an ultra-high performance liquid chromatography (UHPLC) system (Nexera UHPLC LC-30A, Shimadzu), and a triple quadrupole mass spectrometer (QTRAP 4000 LC-MS/MS, AB Sciex). Water was purified using a Milli-Q Advantage A10 system (Millipore). Centrifugation was conducted using a refrigerated high-speed centrifuge (5430R, Eppendorf). Metabolite separation was achieved on a Waters ACQUITY UPLC BEH Amide column (1.7 μm, 2.1 × 100 mm).

**Reagents**

LC-MS grade methanol and acetonitrile were purchased from Honeywell. Ammonium acetate and formic acid (FA) were obtained from Sigma-Aldrich.

**Sample Preparation**

(1) A 100 μL aliquot of the sample was transferred into a 1.5 mL microcentrifuge tube. Subsequently, 300 μL of ice-cold acetonitrile containing 0.1% FA was added to the sample, followed by vortexing for 2 min.

(2) The mixture was centrifuged at 13,000 × g and 4°C for 15 min. The supernatant was then carefully transferred to a new 1.5 mL tube. This centrifugation and transfer step was repeated twice to ensure complete protein precipitation.

(3) The final supernatant was collected for direct injection into the LC-MS/MS system.

(4) Quality control (QC) samples were prepared by pooling equal volumes from each individual sample to monitor instrument stability throughout the analysis.

**LC-MS/MS Conditions**

Chromatographic Conditions: Metabolite separation was performed on an ACQUITY UPLC BEH Amide column (1.7 μm, 2.1 mm × 100 mm) at a column temperature of 40°C. The mobile phase consisted of Solvent A (water containing 5 mM ammonium acetate and 0.1% FA) and Solvent B (acetonitrile). The flow rate was set at 0.3 mL/min with an injection volume of 5 μL. The autosampler was maintained at 10°C. The gradient elution program was as follows: 0–1 min, 90% B; 1–8 min, linear gradient from 90% B to 50% B; 8–8.1 min, return to 90% B; 8.1–10 min, re-equilibration at 90% B. The total run time was 10 min.

Mass Spectrometry Conditions: Data acquisition was performed using the AB SCIEX QTRAP™ 4000 LC-MS/MS system equipped with an electrospray ionization (ESI) source in positive ion mode. The key MS parameters were optimized as follows: Ion spray voltage (ISVF): +5500 V; Nebulizer gas (Gas 1): 60 psi; Heater gas (Gas 2): 60 psi; Curtain gas (CUR): 35 psi; Ion source temperature: 600°C. Quantification was executed using the Multiple Reaction Monitoring (MRM) mode.

**Molecular Docking Analysis**

Molecular docking was performed to predict the binding modes and affinities between the target proteins (CutC, TMM, and FMO3) and the ligands (Til, Aca, and Aca-7-G). The amino acid sequences and structural information of the target proteins were retrieved from the **UniProt database** ([https://www.uniprot.org/](https://www.google.com/url?sa=E&q=https%3A%2F%2Fwww.uniprot.org%2F)) and the **RCSB Protein Data Bank** ([https://www.rcsb.org/](https://www.google.com/url?sa=E&q=https%3A%2F%2Fwww.rcsb.org%2F)).

Protein preparation was conducted using **Discovery Studio 2019**, which involved the removal of crystallographic water molecules, addition of hydrogen atoms, assignment of formal charges, and repair of missing residues or side chains. The optimized protein structures were exported in PDB format. The 3D structures of the small molecule ligands were obtained from the **PubChem database** and subjected to energy minimization using the CHARMm force field within Discovery Studio 2019.

The prepared protein and ligand files were converted to the PDBQT format using **AutoDockTools 4.2**. Molecular docking was executed using **AutoDock Vina 1.2.6** with a grid box centered on the known active sites or cofactor-binding pockets (e.g., FAD/NADP binding regions), ensuring a search space large enough to accommodate the ligands. The docking results were ranked by their binding affinities (kcal/mol), and the conformation with the lowest binding energy was selected for further analysis. Visualization of the 3D binding models and 2D ligand-protein interaction diagrams was performed using **PyMOL 3.1** and **Discovery Studio 2019**.

**Supplementary Figures**

**Figure S1** **Tilianin alleviates atherosclerotic burden and promotes plaque stability in a dose-dependent manner.**

(A) Representative images of en face Oil Red O (ORO) staining of the whole aorta from each group (Til-L: 2.1 mg/kg/d, Til-M: 3.5 mg/kg/d, Til-H: 7 mg/kg/d, Simvastatin: 3.5 mg/kg/d). (B) Quantitative analysis of the aortic lesion area expressed as a percentage of the total aortic surface area. (C) Representative histological sections of the aortic root stained with Hematoxylin and Eosin (HE), Masson’s trichrome, and ORO. Black dashed lines delineate the atherosclerotic plaque areas. Scale bars = 200μm. (D) Quantification of the necrotic core area expressed as a percentage of the total plaque area (calculated from HE sections). (E) Quantification of the collagen-positive area (blue staining) within the plaque area (calculated from Masson’s trichrome sections). (F) Quantification of the cross-sectional lesion area in the aortic root expressed as a percentage of the total vessel area (calculated from ORO sections).

Data are expressed as mean ± SD. ^#^P > 0.05 vs. Control. *P < 0.05 vs. Model. ***P* < 0.01 vs. Model. ****P* < 0.001 vs. Model.

**Figure S2** **Fecal colony counts in conventional and pseudo-germ-free (PGF) mice.**

Data are presented as mean ± SD (n = 6). **P* < 0.05.

**Figure S3. Effect of gut microbiota on the pharmacokinetics of Til and its metabolites.**

(A) Plasma concentration–time profiles of Til, Aca, and Aca-7-G after oral administration of Til (21 mg/kg) in conventional and PGF mice. Data are presented as mean ± SD (n = 6). *P < 0.05. (B) AUC*_0-t_* of Til, Aca, and Aca-7-G after oral administration of Til (21 mg/kg) in conventional and PGF mice. Data are presented as mean ± SD (n = 6). **P* < 0.05.

**Figure S4 A single intragastric administration of Til can significantly reduce the plasma TMAO level in mice.**

**Figure S5 Specific Chromatograms of Tilianin, Acacetin and Aca-7-G in Mouse Plasma.**

(A) Blank plasma; (B) Blank plasma spiked with standards; (C) Plasma sample collected after drug administration in mice.

**Figure S6 Representative Total Ion Chromatograms (TICs) for TMA and TMAO analysis.**

Representative TICs obtained from the LC-MS/MS analysis of three overlaid Quality Control (QC) samples. The target metabolites were well-separated on a Waters ACQUITY UPLC BEH Amide column. Key peaks are identified by their retention times (RT): TMA (RT = 3.25 min) and TMAO (RT = 4.38 min). The high degree of overlap among the three QC samples indicates excellent instrumental stability and methodological reproducibility.

**Figure S7 Representative LC-MS/MS chromatograms of short-chain fatty acids (SCFAs).**

The overlaid Total Ion Chromatograms (TICs) display the separation of seven SCFAs, resolved as five chromatographic groups, and the internal standard in fecal samples using an LC-MS/MS-based derivatization method. The target analytes were well-separated within a 10-minute run. The identified peaks based on their mass-to-charge ratios and retention times (RT) are: (1) Acetic acid (3.08 min); (2) Propanoic acid (3.26 min); (3) Butyric/Isobutyric acid (3.45 min); (4) Valeric/Isovaleric acid (3.74 min); (5) Hexanoic acid (4.39 min); and (6) Internal Standard (5.90 min). The high degree of peak alignment across multiple injections ensures the high sensitivity and precision of the SCFA quantification.

**Supplementary Tables**

**Table S1 Pharmacokinetic parameters of Til and its metabolites in mice after oral administration of a low dose of Til (7 mg/kg) (Mean ± SD, n=6)**

| **Parameters** | **Til** | | **Aca** | | **Aca-7-G** | |
| --- | --- | --- | --- | --- | --- | --- |
|  | **Control** | **PGF** | **Control** | **PGF** | **Control** | **PGF** |
| ***T_max_* (min)** | 12.5 ± 9.8 | 47.5 ± 65.3 | 205.8 ± 146.1 | 132.5 ± 130.9 | 152.5 ± 161.5 | 106.6 ± 149.8 |
| ***C_max_* (μg/L)** | 13.6 ± 19.1 | 16.1 ± 8.3 | 10.5 ± 6.1 | 7.0 ± 8.2 | 119.1 ± 88.1 | 168.1 ± 124.1 |
| ***AUC _(0-t)_* µg·min/L** | 870.3 ± 654.3 | 3447.2 ± 1352.0* | 1267.1 ± 827.2 | 918.4 ± 1024.2 | 26211.7 ± 12334.2 | 38158.3 ± 21544.9 |
| ***AUC _(0-∞)_* µg·min/L** | 1732.1 ± 1557.2 | 3591.8 ± 1369.7* | 1912.1 ± 1503.1 | 1527.3 ± 1864.2 | 54786.5 ± 27003.8 | 101567.4 ± 44109.6 |
| ***T_1/2_*（min）** | 95.5 ± 85.4 | 147.9 ± 170.5 | - | - | - | - |

**Table S2 Pharmacokinetic parameters of Til and its metabolites in mice after oral administration of a high dose of Til (21 mg/kg) (Mean ± SD, n=6)**

| **Parameters** | **Til** | | | **Aca** | | **Aca-7-G** | |
| --- | --- | --- | --- | --- | --- | --- | --- |
|  | **Control** | **PGF** | **Control** | | **PGF** | **Control** | **PGF** |
| ***T_max_* (min)** | 56.6 ± 65.2 | 282.5 ± 344.2 | 70.8 ± 57.8 | | 13.3 ± 2.8 | 20.0 ± 21.9 | 9.2 ± 10.2 |
| ***C_max_* (μg/L)** | 16.3 ± 12.9 | 29.5 ± 26.9 | 10.8 ± 8.2 | | 14.1 ± 9.4 | 82.7 ± 56.2 | 220.8 ± 154.1 |
| ***AUC _(0-t)_* µg·min/L** | 6301.9 ± 4136.6 | 8588.5 ± 6217.8 | 954.6 ± 1031.8 | | 636.5 ± 102.1 | 13558.1 ± 5179.8 | 44895.5 ± 82966.4 |
| ***AUC _(0-∞)_* µg·min/L** | 11654.1 ± 10555.5 | 33514.9 ± 43174.7 | 1899.3 ± 2105.6 | | 719.9 ± 112.5 | 14135.3 ± 5207.1 | 45764.6 ± 82623.0 |
| ***T_1/2_*（min）** | 1254.2 ± 689.9 | 998.9 ± 850.1 | - | | - | - | - |

**Table S3 Binding affinities (kcal/mol) of Tilianin and its metabolites toward key enzymes in the TMA/TMAO pathway.**

| Target protein | Til | Aca | Aca-7-G |
| --- | --- | --- | --- |
| TMM | -9.3 ± 0.31 | -8.12 ± 0.25 | -9.60 ± 0.32 |
| CutC | -6.8 ± 0.67 | -6.5 ± 0.48 | -7.3 ± 0.39 |
| FMO3 | -8.9 ± 0.28 | -7.8 ± 0.34 | -9.4 ± 0.29 |

*Data are presented as mean ± SD of the top three binding poses.*

**Table S4 Inhibitory potency (IC50) of Til and its metabolites against microbial TMAO production. Values were obtained by four-parameter logistic regression fitted to all individual replicate values (nine concentrations × six biological replicates, 54 points per compound); the 95% confidence intervals are those returned by that fit**

| Compound | *IC*_50_ (nM) | 95% Confidence Interval(nM) | *R*^2^ | Hill slope |
| --- | --- | --- | --- | --- |
| Til | 18.78 | 18.40 to 19.17 | 0.9984 | -1.513 |
| Aca | 34.22 | 33.28 to 35.20 | 0.9968 | -1.609 |
| Aca-7-G | 64.72 | 62.91 to 66.60 | 0.9964 | -1.602 |

**Table S5 In vitro inhibitory effects of Til and its metabolites on microbial TMAO production (TMAO, mean ± SD of six independent biological replicates; relative activity expressed as a percentage of the 0 nM control).**

| Concentration (nM) | Til | | Aca | | Aca-7-G | |
| --- | --- | --- | --- | --- | --- | --- |
|  | Relative Activity (%) Mean ± SD | TMAO (μg/mL)  Mean ± SD | Relative Activity (%) Mean ± SD | TMAO (μg/mL)  Mean ± SD | Relative Activity (%) Mean ± SD | TMAO (μg/mL)  Mean ± SD |
| 0 | 100.0 | 85.2 ± 1.5 | 100.0 | 84.8 ± 1.6 | 100.0 | 85.3 ± 1.6 |
| 1 | 96.4 ± 0.2 | 82.1 ± 1.3 | 98.4 ± 0.3 | 83.5 ± 1.6 | 99.2 ± 1.9 | 84.6 ± 0.1 |
| 5 | 88.5 ± 0.6 | 75.4 ± 1.8 | 95.0 ± 1.8 | 80.8 ± 2.5 | 98.3 ± 1.9 | 83.8 ± 0.1 |
| 10 | 72.5 ± 0.7 | 61.8 ± 1.5 | 91.4 ± 0.9 | 77.5 ± 2.2 | 92.2 ± 1.0 | 78.6 ± 1.0 |
| 20 | 48.4 ± 1.2 | 41.2 ± 1.7 | 70.2 ± 1.8 | 59.6 ± 2.6 | 88.5 ± 1.4 | 75.5 ± 2.6 |
| 35 | 25.6 ± 0.9 | 21.8 ± 1.2 | 46.9 ± 1.7 | 39.8 ± 2.2 | 72.2 ± 2.3 | 61.6 ± 3.1 |
| 75 | 12.2 ± 0.7 | 10.4 ± 0.7 | 22.6 ± 1.4 | 19.2 ± 1.6 | 45.5 ± 1.9 | 38.9 ± 2.4 |
| 150 | 5.8 ± 0.6 | 5.0 ± 0.6 | 10.4 ± 1.2 | 8.8 ± 1.2 | 18.6 ± 1.6 | 15.9 ± 1.6 |
| 500 | 1.5 ± 0.2 | 1.3 ± 0.2 | 3.5 ± 0.5 | 3.0 ± 0.5 | 5.2 ± 0.9 | 4.4 ± 0.8 |

**Table S6 Regression equations, linear ranges, and LLOQs of the three analytes.**

| Analytes | Regression equation | Correlation  coefficient (*r*) | Linear range  (mg/L) | LLOQ  (mg/L) |
| --- | --- | --- | --- | --- |
| Tilianin | Y=0.002 x+0.0025 | 0.9987 | 0.78125-100 | 0.78125 |
| Acacetin | Y = 0.0153 x-0.0052 | 0.998 | 0.78125-100 | 0.78125 |
| Aca-7-G | Y=0.0108 x-0.0227 | 0.9924 | 3.125-400 | 3.125 |

**Table S7 Precision and accuracy for the determination of the three analytes in mice plasma (*n*=3 days, 6 replicates per day).**

| Analytes | Spiked  Conc  (mg/L) | Intra-day (*n*=6) | | |  | Inter-day (*n*=6) | | |
| --- | --- | --- | --- | --- | --- | --- | --- | --- |
|  |  | Measured  conc.*a*(mg/L) | Precision  (RSD, %) | Accuracy  (RE, %) | | Measured  conc.*a*(mg/L) | Precision  (RSD, %) | Accuracy  (RE, %) |
| Tilianin | 2 | 2.1 ± 0.3 | 11.99 | 10.1 | | 2.2 ± 0.3 | 13.4 | 9.2 |
|  | 10 | 11.4 ± 0.9 | 7.8 | 14.1 | | 11.4 ± 0.9 | 7.7 | 14.3 |
|  | 80 | 79.4 ± 2.8 | 3.6 | -0.8 | | 79.8 ± 2.5 | 3.1 | -0.33 |
| Acacetin | 2 | 1.8 ± 0.1 | 4.03 | -7.1 | | 1.8 ± 0.1 | 4.5 | -8.2 |
|  | 10 | 10.2 ± 0.3 | 3.1 | 2.0 | | 10.1 ± 0.4 | 4.4 | 0.504 |
|  | 80 | 78.3 ± 3.4 | 4.3 | -2.1 | | 78.3 ± 3.48 | 4.5 | -2.07 |
| Aca-7-G | 9 | 8.6 ± 0.2 | 2.26 | -3.9 | | 9.1 ± 0.5 | 5.2 | 0.7 |
|  | 50 | 50.3 ± 1.4 | 2.8 | 0.6 | | 49.2 ± 3.2 | 6.4 | -1.6 |
|  | 300 | 307.9 ± 12.5 | 4.1 | 2.6 | | 301.4 ± 22.4 | 7.4 | 0.47 |

**Table S8 Stability of the three analytes in mice plasma (*n*=3).**

| Analytes | Spiked  Conc  (mg/L) | 3 freeze-thaw cycles  stability | | Room temperature  stability for 12 h | | Long-term stability  (-20 ℃ for 14 d) | |
| --- | --- | --- | --- | --- | --- | --- | --- |
|  |  | Precision  (RSD, %) | Accuracy  (RE, %) | Precision  (RSD, %) | Accuracy  (RE, %) | Precision  (RSD, %) | Accuracy  (RE, %) |
| Tilianin | 2 | 2.03 | 3.78 | 9.32 | 13.31 | 13.88 | 8.22 |
|  | 10 | 6.99 | 8.66 | 7.95 | 5.95 | 8.09 | 9.52 |
|  | 80 | 1.81 | -2.65 | 2.66 | 0.24 | 2.37 | -0.97 |
| Acacetin | 2 | 4.9 | -8.9 | 2.3 | -11.3 | 5.1 | -4.6 |
|  | 10 | 2.1 | 0.6 | 2.6 | 2.2 | 5.9 | 0.02 |
|  | 80 | 4.9 | 0.1 | 4.1 | -1.1 | 2.2 | -0.4 |
| Aca-7-G | 9 | 1.89 | -2.5 | 1.2 | -1.1 | 11.6 | -2.1 |
|  | 50 | 3.5 | -1.9 | 5.6 | 2.2 | 4.6 | 5.8 |
|  | 300 | 7.6 | 2.3 | 1.78 | 3.9 | 4.1 | 5.3 |

**Table S9 Mean extraction recoveries and matrix effects of the three analytes (*n*=6).**

| Analytes | Spiked Conc  (mg/L) | Mean extraction recovery (%) | Matrix effect (%) |
| --- | --- | --- | --- |
| Tilianin | 2 | 79.8(5.9) | 91.8(3.7) |
|  | 10 | 85.2(7.4) | 85.7(8.6) |
|  | 80 | 86.6(3.8) | 81.4(5.5) |
| Acacetin | 2 | 88.6(5.3) | 97.6(9.8) |
|  | 10 | 93.9(2.3) | 89.7(7.5) |
|  | 80 | 89.3(4.7) | 102.9(5.8) |
| Aca-7-G | 9 | 81.5(2.6) | 96.8(7.5) |
|  | 50 | 83.8(8.7) | 73(9.0) |
|  | 300 | 103.8(5.2) | 106.9(4.7) |

**Table S10 Regression equations and linear ranges of the five short-chain fatty acid analytes.**

| Analytes | Regression equation | Correlation  coefficient (*r*) | Linear range  (mg/L) |
| --- | --- | --- | --- |
| Acetic acid | Y = 9E-09x - 0.0001 | 0.9999 | 0.1-100 |
| Propanoic acid | Y = 6E-08x - 0.0003 | 0.9983 | 0.1-100 |
| Butyric acid+Isobutyric acid | Y = 2E-09x - 0.0008 | 0.9906 | 0.1-100 |
| Valeric acid+Isovaleric acid | Y = 2E-09x - 0.0007 | 0.995 | 0.1-100 |
| Hexanoic acid | Y = 4E-09x - 0.0016 | 0.9996 | 0.1-100 |

**Table S11 Regression equations and linear ranges of TMA and TMAO.**

| Analytes | Regression equation | Correlation  coefficient (*r*) | Linear range  (mg/L) |
| --- | --- | --- | --- |
| TMA | Y=2136 x+67478 | 0.9965 | 0.1-2000 |
| TMAO | Y = 1145947 x-288999 | 0.9961 | 0.78125-100 |

**Table S12 Composition of the high-fat, high-cholesterol diet (H10540; Beijing HFK Bioscience Co., Ltd.; formulated with reference to Research Diets D12108C) used for AS model induction.**

| **Ingredient** | **Amount (g/kg diet)** | **Energy (kcal/kg diet)** |
| --- | --- | --- |
| Casein | 222.88 | 891.52 |
| L-Cystine | 3.34 | 13.36 |
| Corn starch | 236.25 | 945.00 |
| Maltodextrin | 79.12 | 316.48 |
| Sucrose | 125.93 | 503.72 |
| Cellulose | 55.72 | 0 |
| Soybean oil | 27.86 | 250.74 |
| Cocoa butter | 172.73 | 1554.57 |
| Mineral mix M1002 | 11.14 | 0 |
| Calcium hydrogen phosphate | 14.49 | 0 |
| Calcium carbonate | 6.13 | 0 |
| Potassium citrate | 18.39 | 0 |
| Vitamin mix V1001 | 11.14 | 46.24 |
| Choline bitartrate | 2.23 | 0 |

Table S12 continued

| **Ingredient** | **Amount (g/kg diet)** | **Energy (kcal/kg diet)** |
| --- | --- | --- |
| Cholesterol | 12.54 | 0 |
| FD&C Blue dye | 0.0056 | 0 |
| FD&C Yellow dye | 0.0056 | 0 |
| **Total** | **1000** | **2521.63** |

Macronutrient composition (mass basis / energy basis): protein 23% / 20%; carbohydrate 45% / 40%; fat 20% / 40%. Total energy density: 4.52 kcal/g. Cholesterol content: 1.254% (w/w; 12.54 g/kg diet). Fat sources: cocoa butter and soybean oil.
